# Epigenetic Reprogramming of the Type III Interferon Response Potentiates Antiviral Activity and Suppresses Tumor Growth

**DOI:** 10.1371/journal.pbio.1001758

**Published:** 2014-01-07

**Authors:** Siyuan Ding, William Khoury-Hanold, Akiko Iwasaki, Michael D. Robek

**Affiliations:** 1 Department of Pathology, Yale University School of Medicine, New Haven, Connecticut, United States of America; 2 Microbiology Graduate Program, Yale University School of Medicine, New Haven, Connecticut, United States of America; 3 Department of Immunobiology, Yale University School of Medicine, New Haven, Connecticut, United States of America; 4 Howard Hughes Medical Institute, Yale University School of Medicine, New Haven, Connecticut, United States of America; Whitehead Institute, United States of America

## Abstract

Type III interferon (IFN-λ) exhibits potent antiviral activity similar to IFN-α/β, but in contrast to the ubiquitous expression of the IFN-α/β receptor, the IFN-λ receptor is restricted to cells of epithelial origin. Despite the importance of IFN-λ in tissue-specific antiviral immunity, the molecular mechanisms responsible for this confined receptor expression remain elusive. Here, we demonstrate that the histone deacetylase (HDAC) repression machinery mediates transcriptional silencing of the unique IFN-λ receptor subunit (IFNLR1) in a cell-type-specific manner. Importantly, HDAC inhibitors elevate receptor expression and restore sensitivity to IFN-λ in previously nonresponsive cells, thereby enhancing protection against viral pathogens. In addition, blocking HDAC activity renders nonresponsive cell types susceptible to the pro-apoptotic activity of IFN-λ, revealing the combination of HDAC inhibitors and IFN-λ to be a potential antitumor strategy. These results demonstrate that the type III IFN response may be therapeutically harnessed by epigenetic rewiring of the IFN-λ receptor expression program.

## Abstract 2

The tissue-specific IFN-λ receptor expression program can be epigenetically remodeled via HDAC inhibition to strengthen anti-viral and anti-tumor activities in the central nervous system.

## Author Summary

Interferons (IFNs) are important cytokines that protect the host from a broad spectrum of pathogens. IFN-λ, the most recently identified IFN, exhibits potent antiviral activity similar to IFN-α/β. In contrast to the ubiquitous presence of the IFN-α/β receptor, however, the expression of the IFN-λ receptor is restricted to epithelial cells and hepatocytes and absent from immune cells and brain cells. The molecular mechanism responsible for this tissue-specific IFN-λ receptor expression has been elusive. In this study, we demonstrate that histone acetylation mediates chromatin relaxation and transcriptional activation of IFN-λ receptor expression in a cell-type-specific manner. When IFN-λ nonresponsive cells—those that don't normally express the IFN-λ receptor—are treated with inhibitors that block histone deacetylase (HDAC) function, these cells express elevated levels of the IFN-λ receptor and gain sensitivity to IFN-λ. Importantly, rewiring the receptor expression program in this manner via use of HDAC inhibitors can protect formerly IFN-λ-insensitive cells, including primary human astrocytes, from both DNA and RNA virus infection. Additionally, treatment with HDAC inhibitors can also render previously nonresponsive cell types susceptible to the antitumor activity of IFN-λ, thereby inducing apoptosis in glioblastoma cells. This work suggests that a combination of small-molecule HDAC inhibitors and IFN-λ could be a potential antiviral and anticancer strategy.

## Introduction

Interferons (IFNs) constitute an indispensable part of the innate immune response. Type I IFNs protect against viral infections, whereas type II IFN is essential for host defense against bacterial and parasitic pathogens. More recently, a new class of cytokines collectively known as the type III IFNs has been identified [1],[2]. In humans, the type III IFN family consists of IFN-λ1, λ2, and λ3 (encoded by *IFNL1*, *IFNL2*, and *IFNL3*). Similar to type I IFNs, IFN-λs are induced upon virus infection, activate IFN-stimulated gene (ISG) expression, and display broad-spectrum antiviral activity [3]–[5]. Recent studies have demonstrated a critical role of IFN-λ in protection against viral infection at epithelial and mucosal surfaces including the lung and gut [6]–[8]. In addition, single nucleotide polymorphisms in *IFNL3* are strongly associated with viral clearance in patients with chronic hepatitis C virus infection [9]–[12], highlighting the importance of this cytokine family in the intrahepatic immune response. Despite its significance, how IFN-λ activity is regulated remains unresolved.

In contrast to the ubiquitously expressed IFN-α/β receptor, the IFN-λ receptor is composed of the unique α chain (encoded by *IFNLR1*) and the IL-10 receptor β chain (encoded by *IL10RB*), and is restricted to epithelial cells and hepatocytes. We and others have previously reported that IFNLR1 but not IL10RB is differentially expressed in various cell types [13]–[16]. IFNLR1 mRNA expression is high in the lung, gut, and liver, and low in other tissues including the brain and most immune cell types, consistent with the observation that IFN-λ primarily protects the epithelium but not the central nervous system (CNS) from virus infection [7],[15]. Despite the pivotal role of IFN-λ in tissue-specific immune responses, nothing is known about the molecular mechanisms that underlie its distinctive receptor expression pattern.

Here, we demonstrate that IFN-λ receptor expression is mechanistically regulated by coordinated epigenetic modifications and transcription factors (TFs) in a cell-type-specific fashion. Importantly, remodeling of *IFNLR1* promoter chromatin by HDAC inhibition increases accessibility to transcription activators and enhances receptor expression in previously nonresponsive cells, rendering them sensitive to the antiviral and antiproliferative activities of IFN-λ. We present the first case that the type III IFN response can be positively harnessed through epigenetic reprogramming of its receptor expression, thereby contributing to viral clearance and tumor growth suppression.

## Results

### IFN-λ Receptor Expression Is Inversely Correlated with *IFNLR1* Promoter Methylation

**Figure 1:**
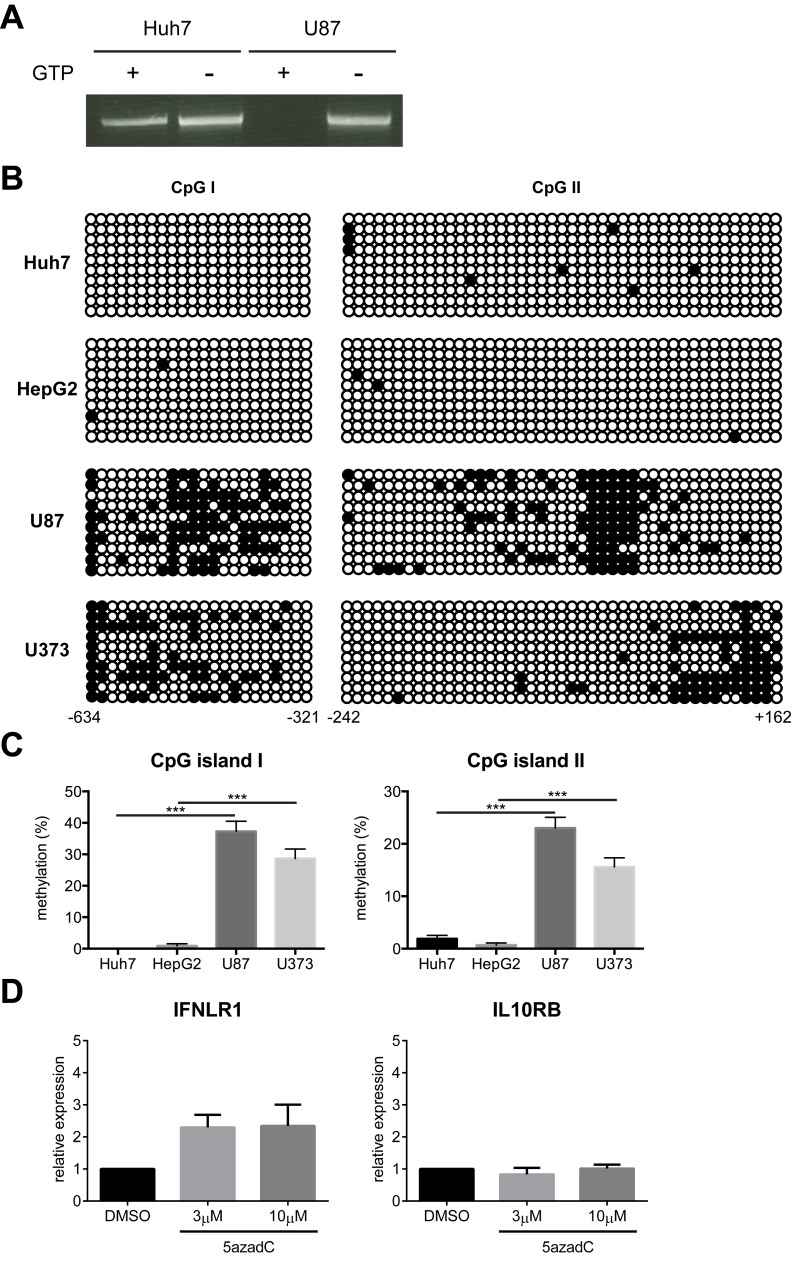
*IFNLR1* promoter methylation negatively correlates with IFN-λ responsiveness. (A) Huh7 and U87 genomic DNA was digested with *McrBC* in the presence or absence of GTP and used as template for nested PCR with primers specific for the *IFNLR1* promoter. (B) Huh7, HepG2, U87, and U373 genomic DNA was subject to bisulfite conversion sequencing. Each circle represents one CpG dinucleotide, with filled circles indicating methylated motifs and open circles nonmethylated motifs. Each row represents an individual clone of the population. Lower numbers indicate relative distance to the TSS. (C) Quantification of the methylation status on both CpG islands in (B). (D) U87 cells were cultured in the presence of vehicle control DMSO, 3 µM, or 10 µM 5azadC for 72 h. IFNLR1 and IL10RB expression was examined by RT-qPCR. In all panels, data represent the mean and standard error of the mean (SEM) of at least three experiments.

Contrary to the broad expression of IL10RB, IFNLR1 is predominantly expressed in cells of epithelial origin. A high level of IFNLR1 mRNA is found in primary human hepatocytes (PHHs), whereas little is detected in primary human astrocytes and neurons [14]. We also observed hypersensitivity to IFN-λ in liver hepatocyte-derived cell lines such as Huh7 and HepG2, in contrast to low responsiveness in brain glia-derived cell lines such as U87 and U373 [14]. To determine the mechanism underlying the cell-type-specific expression pattern, we first investigated the role of epigenetic modifications in this process. Cpgplot analysis of the −3000 to +1000 genomic DNA region relative to the putative *IFNLR1* promoter transcription start site (TSS) identified two adjacent CpG islands (Figure S1A). In the mammalian genome, DNA methylation occurs on the cytosine residues within CpG dinucleotides via the action of DNA methyltransferases (DNMTs). CpG islands are frequently subject to methylation for tissue-specific gene regulation [17]. Therefore, we examined *IFNLR1* promoter methylation in Huh7 and U87 cells using *McrBC*, an endonuclease that specifically cleaves DNA containing methylcytosine in a GTP-dependent manner. We found that the *IFNLR1* promoter was more methylated in U87 cells than Huh7 cells (Figure 1A). To further quantitatively characterize the DNA methylation status, we performed bisulfite conversion sequencing. The *IFNLR1* promoter in Huh7 and HepG2 cells exhibited hypomethylation in contrast to hypermethylation at both CpG islands in U87 and U373 cells (Figure 1B–C), revealing a strong correlation between the DNA methylation pattern and IFNLR1 expression level. Consistently, we also found low levels of DNA methylation in the *IFNLR1* promoter in PHHs (Figure S1B–C). We then asked whether the reversal of methylation would enhance IFNLR1 expression. U87 cells were cultured in the presence of 5-aza-2′-deoxycytidine (5azadC), a nucleoside analogue that potently inhibits DNMT activity. Despite significant reduction of DNA methylation in the *IFNLR1* promoter in 5azadC-treated U87 cells (Figure S1D–E), there was only a marginal increase in IFNLR1 mRNA (Figure 1D), suggesting DNA demethylation alone was insufficient to reactivate transcription.

### Histone Deacetylases Mediate IFN-λ Receptor Silencing in Nonresponsive Cells

**Figure 2:**
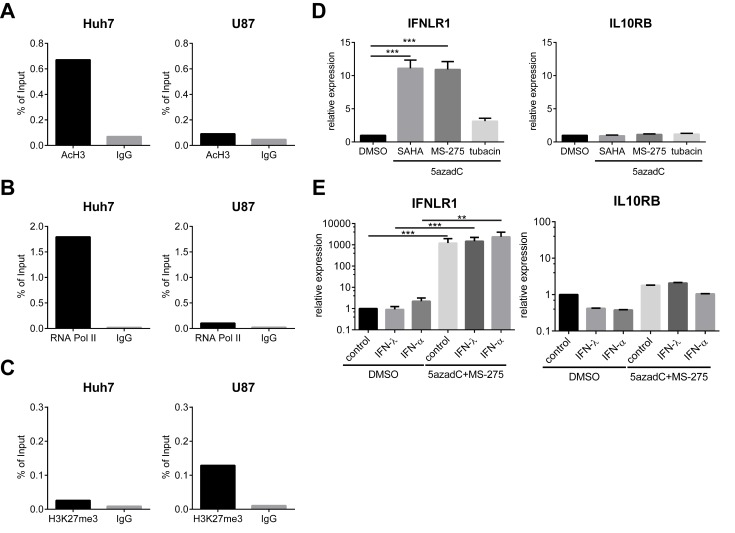
IFNLR1 expression is silenced via HDAC-mediated repression and reactivated by 5azadC and MS-275 treatment. (A–C) ChIP analysis was performed on the *IFNLR1* promoter in Huh7 and U87 cells with AcH3, RNA Pol II, H3K27me3, and control IgG antibodies. (D) U87 cells were cultured with DMSO or 10 µM 5azadC for 72 h. For the latter, 2 µM SAHA, 1 µM MS-275, or 2 µM tubacin were added in the last 24 h. IFNLR1 and IL10RB expression was determined by RT-qPCR. (E) Primary astrocytes were cultured in the presence of DMSO or 10 µM 5azadC (72 h) and 1 µM MS-275 (24 h), and stimulated with PBS, 100 ng/ml IFN-λ1, or 500 U/ml IFN-α for 24 h. IFNLR1 and IL10RB expression was measured by RT-qPCR. In all panels, data represent the mean and SEM of at least three experiments.

In addition to DNA methylation, histone posttranslational modifications including acetylation and methylation also influence chromatin structure and gene expression. Closed chromatin configuration, characteristic of histone H3 trimethyl lysine 9 (H3K9me3) and lysine 27 (H3K27me3), is linked to transcriptional repression. In contrast, acetylation of histone H3 and H4 is associated with active promoters. Strikingly, revealed by chromatin immunoprecipitation (ChIP) using anti-acetylated H3 (AcH3) or control immunoglobulin G (IgG) antibodies, the histone acetylation levels within the *IFNLR1* promoter were considerably higher in Huh7 than U87 cells (Figure 2A). RNA Polymerase II (RNA Pol II), a core component of the transcription complex, was also significantly enriched at the *IFNLR1* promoter in Huh7 cells compared to U87 cells (Figure 2B). Conversely, enrichment of H3K27me3 was only observed in U87 cells, but not Huh7 cells (Figure 2C). These data collectively demonstrate that the *IFNLR1* promoter chromatin structure is relaxed in Huh7 cells in contrast to a more condensed state in U87 cells. Another hallmark of constitutive heterochromatin, H3K9me3, was negative in both cell types (Figure S2A). Importantly, the *IFNLR1* promoter was also hypoacetylated in primary human astrocytes (Figure S2B), which like U87 and U373 cells, displays low levels of IFN-λ receptor expression [14].

Since histone acetylation is controlled by the balance of histone acetyltransferases (HATs) and deacetylases (HDACs), we next asked whether blocking HDAC function would reactivate IFN-λ receptor expression in nonresponsive cells. Consistent with a previous report [18], SAHA and MS-275 increased global H3 acetylation (Figure S2C). IFNLR1 expression was significantly up-regulated in U87 cells when nonspecific HDAC inhibitors such as SAHA, Trichostatin A, and sodium butyrate were used together with 5azadC (Figures 2D and S2D). Importantly, MS-275, a specific inhibitor of HDAC1, also increased IFN-λ receptor expression (Figure 2D). Knockdown of HDAC1 using small interfering RNA (siRNA) further confirmed its role in IFNLR1 silencing (Figure S2E–F). In addition, functional disruption of HDAC2/3 activity by apicidin suggests the involvement for other class I HDACs in the repression machinery as well (Figure S2D). In contrast, IFNLR1 expression was not altered by tubacin, which targets HDAC6, or nicotinamide, a pan-inhibitor for class III HDACs (Figures 2D and S2D). Of note, IFNLR1 expression was also markedly up-regulated in primary astrocytes post-5azadC and -MS-275 treatment (Figure 2E). IL10RB expression was not affected by any of these treatments (Figure 2E), and no increase in IFNLR1 expression was observed in inhibitor-treated Huh7 and A549 cells, both of which have naturally high IFN-λ receptor levels (Figure S2G–H).

To further test whether HDAC-mediated IFNLR1 silencing is a unique mechanism in the brain, we applied inhibitors to a variety of IFN-λ-insensitive cell types. Receptor expression was also up-regulated in Jurkat, BNL, and NIH3T3 cells (Figure S2I–K), suggesting a common repressive mechanism that is not tissue- or species-specific. IFNLR1 expression was also markedly increased in primary human CD4^+^ T cells following the inhibitor treatment (Figure S2L). Taken together, these results indicate that IFNLR1 expression is epigenetically silenced via HDAC-mediated repression in nonresponsive cell types.

### NF-Y, an Activating TF, Differentially Binds to the *IFNLR1* Promoter

**Figure 3:**
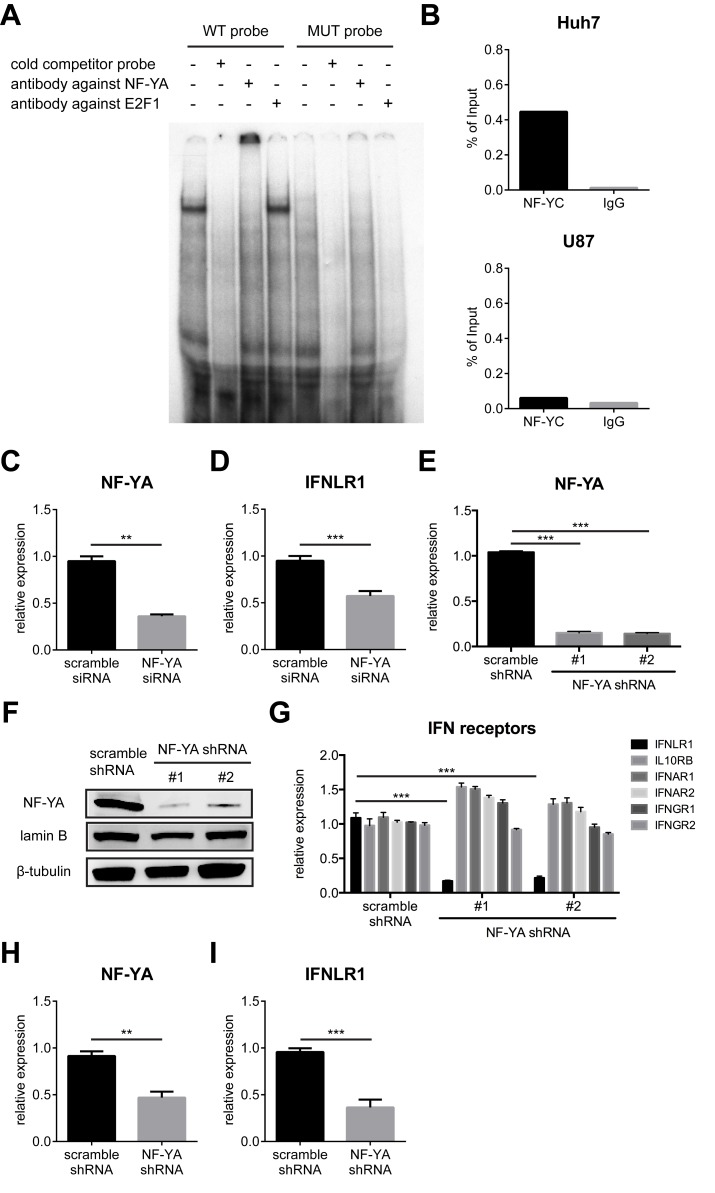
NF-Y binds to the *IFNLR1* promoter sequence and positively regulates IFN-λ receptor expression. (A) Gel mobility shift assay was performed using the wild-type (WT) probe (the −434∼−401 region of the *IFNLR1* promoter) and the mutant (MUT) probe (CCAAT motif substituted with AACCG), incubated with U87 cell nuclear extracts, poly (dA∶dT), 100-fold excess of cold probe, and the indicated antibodies. (B) ChIP analysis was performed on the *IFNLR1* promoter in Huh7 and U87 cells with NF-YC and control IgG antibodies. (C–D) NF-YA and IFNLR1 expression was examined by RT-qPCR in Huh7 cells transfected with scrambled or NF-YA-specific siRNAs. (E, G) Expression of NF-YA and receptor subunits of type I, II, and III IFNs was examined by RT-qPCR in Huh7 cells stably expressing scrambled or NF-YA-specific shRNAs. (F) Lysates from Huh7 stable cell lines were harvested and used for WB using the indicated antibodies. Lamin B serves as loading control for nuclear proteins. (H–I) NF-YA and IFNLR1 expression was measured by RT-qPCR in PHHs infected with lentiviruses encoding scrambled or NF-YA-specific shRNAs. In all panels, data represent the mean and SEM of at least three experiments.

To identify the key TFs that regulate IFNLR1 expression, we performed electrophoretic mobility shift assays (EMSAs) using the −500 to −401 region of the *IFNLR1* promoter. Based on bioinformatics prediction, this region was likely to contain binding sites for a number of TFs with important regulatory functions (Figure S3A). Specific TF binding was observed in the −434 to −401 segment, but not the other areas of this region (Figure S3B). Further mutagenesis revealed that the TF binding motif was located within the −430∼−421 sequences (Figure S3C). *In silico* analysis of the sequence predicts the binding of several candidates including NF-Y and the E2F family proteins (Figure S3A). Antibody supershift confirmed a specific interaction of NF-Y but not E2F1 within this region (Figure 3A). NF-Y is a heterotrimeric TF composed of three subunits. While NF-YA interacts with the sequence specific CCAAT motif, NF-YB and NF-YC directly contact DNA through histone-fold domains [19]. Each base of the CCAAT motif is essential for NF-Y binding, with immediate flanking sequences on both ends also being critical [20]. NF-YA interaction with the *IFNLR1* promoter was verified by the complete loss of binding following mutation of the CCAAT binding site (Figure 3A). We further validated that NF-YB and NF-YC also interacted with the promoter sequence (Figure S4A). In contrast, we did not observe a supershift with another member of the E2F family proteins, E2F4 (Figure S4A). To explore the role of NF-Y in *IFNLR1* transcription, we examined the expression of all three subunits. Surprisingly, none of NF-YA, NF-YB, and NF-YC were discrepantly expressed on both the mRNA and protein levels (Figure S4C–D), implying that NF-Y acts through mechanisms other than differential expression. Although the NF-Y binding site is located within CpG island I, the interaction was independent of DNA methylation (Figure S4B).

Interestingly, ChIP analysis indicated that NF-Y was preferentially bound to the *IFNLR1* promoter in Huh7 cells but not U87 cells, suggesting it to be an activating TF (Figure 3B). To test this hypothesis, NF-Y was silenced using a pool of mixed individual siRNAs. Consistent with our prediction, accompanied with reduced NF-YA expression following siRNA transfection (Figure 3C), IFN-λ receptor expression in Huh7 cells was significantly down-regulated (Figure 3D). Conversely, NF-YA knockdown did not affect IFNLR1 expression in low-expressing U87 cells (Figure S4E–F). In addition to transient depletion, we also generated two stable NF-YA-silenced Huh7 cell lines using lentivirus-delivered short hairpin RNA (shRNA) specifically targeting NF-YA. We verified the specificity of the knockdown (Figure 3E–F) and found pronounced selective down-regulation of IFNLR1, but not IL10RB or type I/II IFN receptor expression (Figure 3G). In contrast, IFNLR1 expression was not altered in low-expressing U373 cells with stable NF-YA knockdown (Figure S4G–H). Importantly, transduction of primary hepatocytes with lentivirus delivering NF-YA-specific shRNA resulted in a simultaneous reduction in both NF-YA and IFNLR1 levels (Figure 3H–I), confirming NF-Y as an activating TF driving IFNLR1 expression in IFN-λ-responsive cells.

### Small-Molecule Inhibitors Relax *IFNLR1* Promoter Chromatin and Increase NF-Y Binding

**Figure 4:**
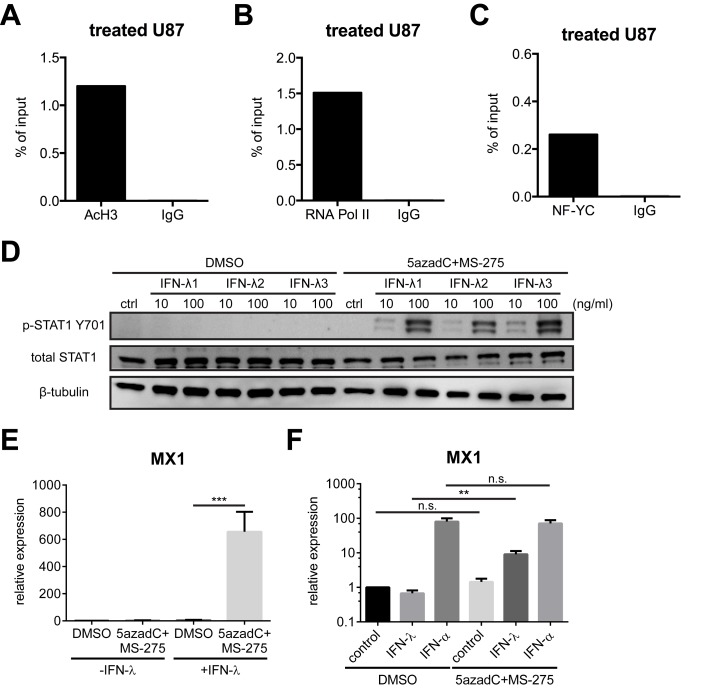
5azadC and MS-275 open *IFNLR1* promoter chromatin and increase IFN-λ sensitivity in nonresponsive cells. (A–C) ChIP analysis was performed on the *IFNLR1* promoter in 5azadC and MS-275-treated U87 cells with AcH3, RNA Pol II, NF-YC, and control IgG antibodies. (D) U87 cells were preincubated with DMSO or small-molecule inhibitors and stimulated with or without 10 ng/ml or 100 ng/ml of IFN-λ1/2/3 for 6 h. Lysates were used for WB using the indicated antibodies. (E–F) MX1 expression was measured by RT-qPCR in U87 cells and primary astrocytes following the indicated treatment. In all panels, data represent the mean and SEM of at least three experiments.

To further investigate the molecular mechanism by which HDAC inhibition enhances IFNLR1 expression, we examined *IFNLR1* promoter chromatin by surveying enrichment of acetylated histones and TFs in inhibitor-treated U87 cells. In contrast to the lack of active transcription signatures prior to treatment (Figure 2A–B), levels of AcH3 and RNA Pol II were substantially elevated (Figure 4A–B). Interestingly, although the expression of NF-Y subunits was not increased by the inhibitors (Figure S5A–B), NF-Y binding affinity to the *IFNLR1* promoter was markedly augmented in U87 cells (Figure 4C), suggesting that the chromatin configuration was reshaped to permit access of activating *trans*-acting factors such as NF-Y.

### Up-Regulated IFN-λ Receptor Expression Restores Sensitivity to IFN-λ and Potentiates Antiviral Activity

**Figure 5:**
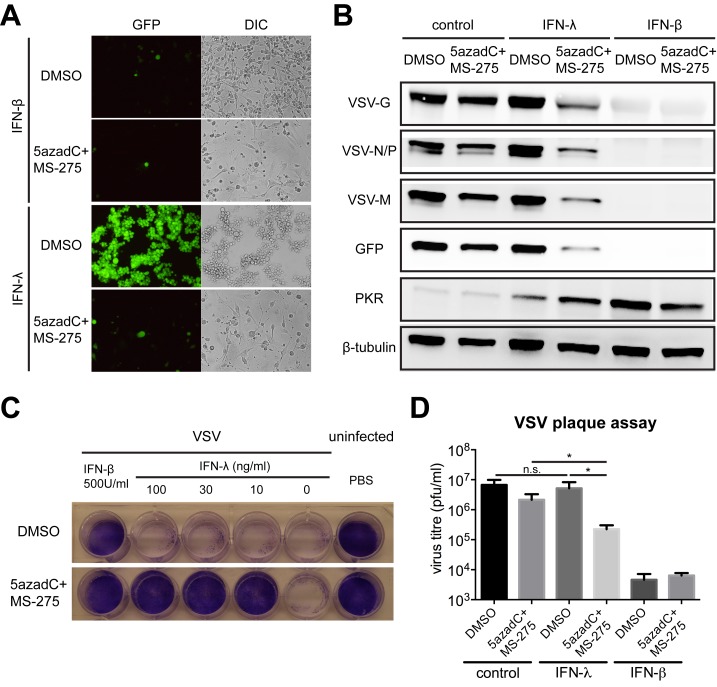
IFN-λ protects U87 cells from VSV infection in the presence of small-molecule inhibitors. (A) U87 cells were treated with DMSO or inhibitors, stimulated with 100 ng/ml IFN-λ1 or 500 U/ml IFN-β for 24 h, and infected with VSV-GFP (MOI = 0.1) for 16 h. GFP expression was monitored by fluorescence microscopy (representative images are shown; 40× magnification). (B) Lysates from infected U87 cells following the indicated treatment were used for WB using indicated antibodies. G, glycoprotein; N, nucleoprotein; P, phosphoprotein; M, matrix protein. (C) Cytolytic effects were measured in U87 cells following the indicated treatment and infection with VSV (MOI = 1) for 24 h. Viable cells were stained with crystal violet. (D) VSV titers in infected U87 cells were determined by a standard plaque assay. In all panels, data represent the mean and SEM of at least three experiments.

To assess the physiological relevance of pharmacologically enhanced receptor expression, we stimulated U87 cells with IFN-λ in the presence or absence of DNMT and HDAC inhibitors, and measured downstream signaling, gene expression, and antiviral activity. IFN-λ signaling was more potently activated in inhibitor-primed U87 cells, revealed by increased STAT1 tyrosine phosphorylation (Figure 4D) and ISG expression (Figures 4E and S5C–E). Importantly, we also observed higher levels of phosphorylated STAT1 and elevated MX1 expression in inhibitor-treated astrocytes in response to IFN-λ (Figures S5F and 4F), whereas IFN-α induced comparable MX1 levels (Figure 4F), implying 5azadC and MS-275 did not affect IFN-α/β receptor expression. To determine if increased ISG induction reinforces antiviral activity, U87 cells were cultured with or without inhibitors, exposed to IFN-λ or IFN-β, and infected with vesicular stomatitis virus (VSV) expressing GFP. IFN-β completely blocked VSV infection, whereas IFN-λ exclusively protected inhibitor-treated U87 cells from VSV (Figure 5A–B). Even at the lowest concentration, IFN-λ counteracted the cytopathic effect (CPE) of VSV infection in inhibitor-treated U87 cells, whereas MS-275 alone did not confer protection (Figure 5C). Furthermore, virus titer from infected U87 cells was decreased in the presence of inhibitors and IFN-λ (Figure 5D).

**Figure 6:**
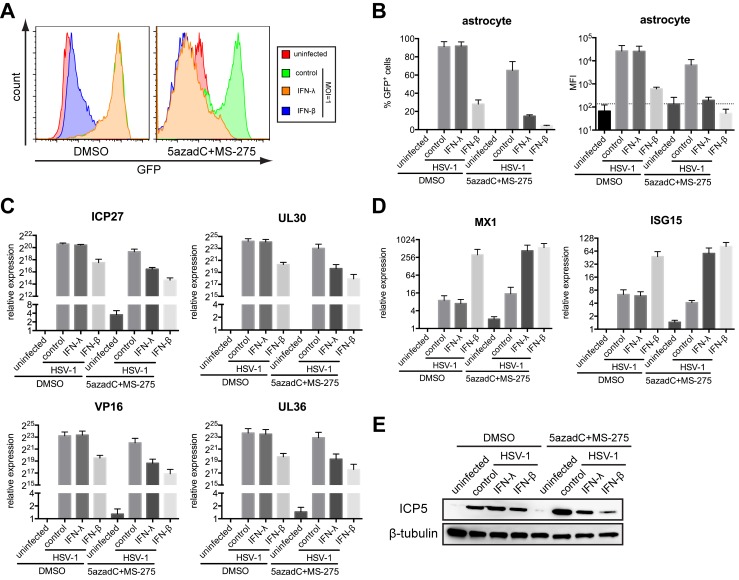
IFN-λ protects primary astrocytes from HSV-1 infection in the presence of small-molecule inhibitors. (A) Primary human astrocytes were treated with DMSO or inhibitors, stimulated with 100 ng/ml IFN-λ1 or 500 U/ml IFN-β for 24 h, and infected with HSV-1-GFP (MOI = 1). At 12 h postinfection, GFP expression was analyzed by flow cytometry. (B) Quantification of the percentage of GFP positive cells and mean fluorescence intensity (MFI) in (A). (C) Expression of HSV-1 genes, including ICP27 (immediate early), UL30 (early), VP16 (leaky-late), and UL36 (true-late), was measured by RT-qPCR. (D) Expression of MX1 and ISG15 was measured by RT-qPCR. (E) Lysates from infected astrocytes were used for WB using indicated antibodies. In all panels, data represent the mean and SEM of at least three experiments.

We next extended our study to primary human astrocytes. Reprogramming the *IFNLR1* promoter sensitized astrocytes to IFN-λ and protected against VSV infection at all MOIs (Figure S6A–C). We further tested the antiviral activity to herpes simplex virus–1 (HSV-1), a clinically important DNA virus that establishes latent infection in the CNS. Without inhibitors, astrocytes were efficiently infected by HSV-1, even in the presence of IFN-λ, suggesting that chromatin remodeling is required for IFN-λ signaling in astrocytes (Figure 6A–B). Notably, we observed less viral gene expression and stronger ISG induction in inhibitor-primed, IFN-λ stimulated astrocytes (Figure 6C–E). We also assessed the efficacy against another neurotropic DNA virus, human cytomegalovirus (HCMV). Consistently, IFN-λ reduced HCMV-GFP infection at all MOIs in inhibitor-treated astrocytes (Figure S7A–C). These results collectively demonstrate that up-regulated IFNLR1 expression in brain cells by inhibitor treatment sensitizes them to IFN-λ signaling and protects against both DNA and RNA virus infections.

### MS-275 and IFN-λ Cooperatively Induce Apoptosis and Suppress Glioblastoma Proliferation

**Figure 7:**
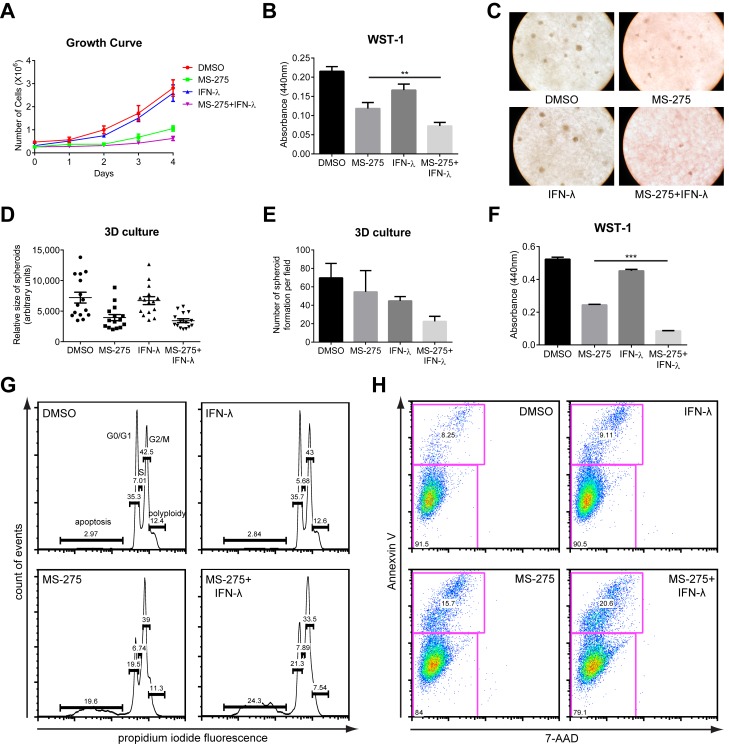
Combination of MS-275 and IFN-λ inhibits cancer proliferation by elevated rate of apoptosis. (A) U87 cells were cultured in the presence of DMSO, 1 µM MS-275 alone, 100 ng/ml IFN-λ1 alone, or both for the course of 4 d. Cell numbers were manually determined by hemacytometer counting at the indicated time points. (B, F) Cell proliferation of U87 cells or U87 spheroids in 3D culture with indicated treatment were performed using the WST-1 assay, which measures active cellular metabolism. (C) U87 spheroid formation in 3D culture was photographed at day 14 in culture (representative images are shown; 200× magnification). (D–E) Quantification of the relative sizes and numbers of U87 spheroids in (C). (G) Cell cycle analysis was performed in U87 cells with indicated treatment using propidium iodide staining. Numbers in the histogram show fractions (percent) of sub-G1, N, 2N, and polyploidy from left to right. (H) U87 cells with indicated treatment were stained with Annexin V-FITC and 7-AAD. Numbers indicate the percentage of FITC-positive cells (upper left quadrant). FITC, fluorescein isothiocyanate; 7-AAD, 7-Aminoactinomycin. In all panels, data represent the mean and SEM of at least three experiments.

In addition to their antiviral functions, type I IFNs are also recognized for their antiproliferative activity, with IFN-α being used clinically in the treatment of multiple myeloma and metastatic melanoma [21]. The antitumor activity of IFN-λ has also been established in multiple cancer models [22]–[24]. However, the potential clinical application of IFN-λ is limited by its restricted receptor distribution. We therefore explored whether IFNLR1 up-regulation by HDAC inhibitors renders cancer cells sensitive to the antitumor properties of IFN-λ. In accordance with our hypothesis, combined treatment of U87 glioblastoma cells with MS-275 and IFN-λ inhibited growth in culture over a 4-d time course, while IFN-λ alone had minimal effects on proliferation (Figure 7A–B). Of note, the combination did not substantially affect the proliferation of primary astrocytes (Figure S8A), likely due to selective effects exerted by MS-275 on rapidly replicating tumor cells. We also employed a three-dimensional spheroid culture system to mimic physiological conditions of tumor growth. Consistently, the combined treatment reduced both the size and number of U87 spheroid formation (Figure 7C–E). WST-1 assay in the 3D culture also revealed reduced glioblastoma proliferation following the combination treatment (Figure 7F).

Many factors may contribute to cancer cell growth inhibition, including senescence, cell cycle arrest, dampened survival signaling, or increased apoptosis. Senescence-associated β-galactosidase (SA-β-gal) activity was comparable in all treatment groups, suggesting that the proliferation defect we observed was independent of senescence (Figure S8B–C). Both mammalian target of rapamycin (mTOR) and NF-κB signaling are linked with cell growth and commonly deregulated in cancers, but no disruption in these pathways was detected following treatment (Figure S8D). Intriguingly, propidium iodide staining revealed an increase in the sub-G1 cell cycle population, indicative of apoptotic cells, in U87 cells with the combined treatment (Figure 7G). Consistently, we found an elevated frequency of Annexin V^+^ cells in combined compared to single treatment, further verifying an affirmative role of apoptosis in growth inhibition (Figure 7H). Stronger activation of the intrinsic cell death pathway, represented by caspases 3 and 9 cleavage, correlated with the increased level of apoptosis with the combined treatment (Figure S8D). In addition to U87 glioblastoma, we extended our study to mouse B16 melanoma, a cancer cell type that responds to mouse IFN-λ (Figure S8E). As expected, either MS-275 or IFN-λ alone led to cell growth inhibition, but the combination again had the greatest effect (Figure S8F). In conclusion, we demonstrate that mechanistically, enhanced receptor expression renders cancer cells susceptible to the pro-apoptotic activity of IFN-λ and thus restricts tumor cell expansion.

## Discussion

**Figure 8:**
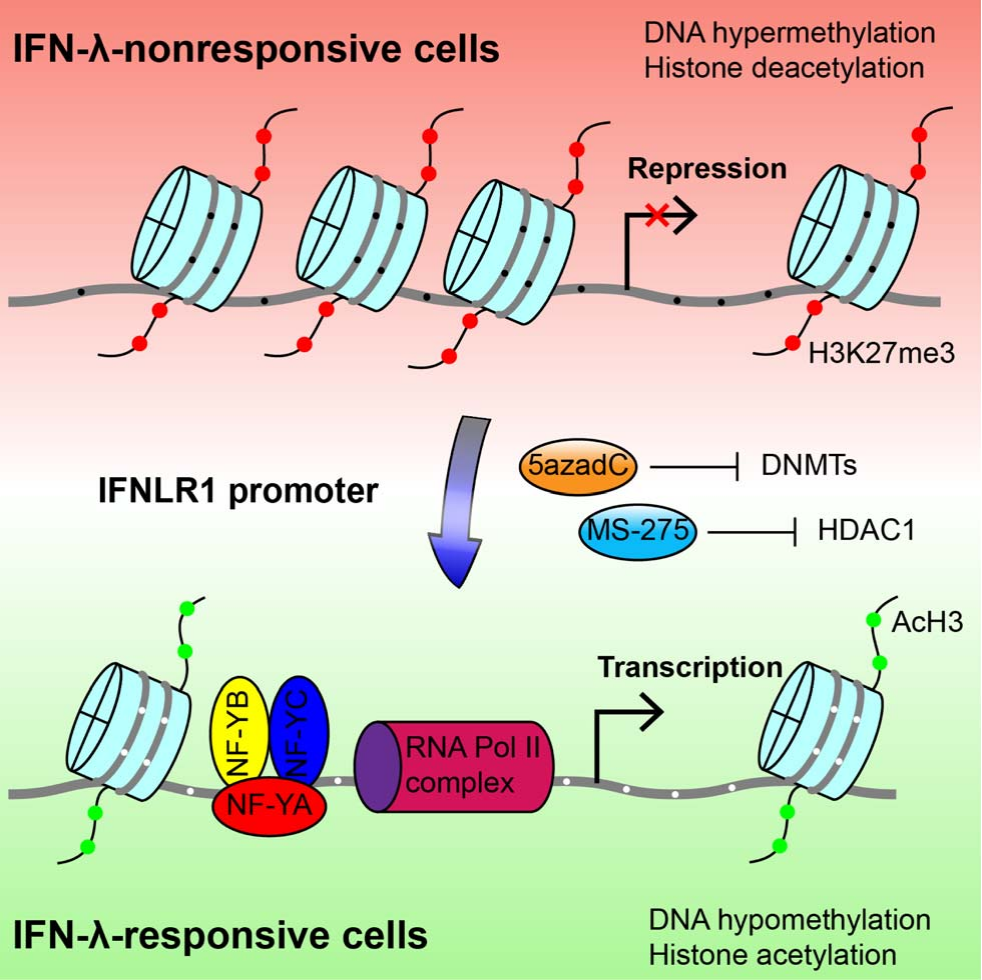
Molecular mechanism of differential IFNLR1 expression. Epigenetic silencing is the major mechanism that restricts IFNLR1 expression in IFN-λ nonresponsive cell types. In the low-expressing cells (red background), the *IFNLR1* promoter is repressed through DNA hypermethylation, histone hypoacetylation, and loss of affinity for TFs and RNA Polymerase II complexes. In contrast, the *IFNLR1* promoter is associated with DNA hypomethylation, histone acetylation, and binding of activating TF (represented by trimeric NF-Y) and RNA Polymerase II in high-expressing cells (green background). Importantly, sensitivity to IFN-λ can be regained by blocking DNMT and HDAC activities with 5azadC and MS-275, which epigenetically reconfigure the promoter chromatin structure. Gray line, *IFNLR1* promoter DNA; red filled circles, H3K27 trimethylation; green filled circles, histone H3 acetylation; black filled circle, methylated CpG dinucleotides; white filled circle, unmethylated CpG dinucleotides.

A central question to IFN-λ regulation is how the restricted distribution of its receptor is achieved. Here, we dissected the role of epigenetic modifications and TFs in determining the tissue-specificity of IFN-λ receptor expression. We identified HDAC-mediated closed chromatin conformation as the major silencing mechanism of IFNLR1 expression in IFN-λ-insensitive cells. In addition, we demonstrated that the *IFNLR1* promoter is exclusively accessible for the transcription activator NF-Y in IFN-λ-responsive cells. Remarkably, 5azadC and MS-275 up-regulate IFN-λ receptor expression and restore IFN-λ sensitivity in previously nonresponsive cell types (Figure 8). Taken together, these findings establish the mechanism underlying the tissue-specific regulation of the IFN-λ response and lay the foundation for the future translational use of the cytokine.

The brain is a partially immune privileged site and protected from most virus infections by effective peripheral clearance and multilayer physical barriers. Unfortunately, some neurotropic viruses including α- and β-herpesviruses, represented by HSV-1 and HCMV, can still breach the defense and infect neurons and astrocytes. Using 5azadC and MS-275, which epigenetically rewire the IFNLR1 expression program in primary astrocytes, we demonstrated that the type III IFN response can be remodeled to protect against both DNA and RNA viruses. Notably, MS-275 is capable of penetrating the blood–brain barrier [25], and therefore has the potential to be used clinically in this respect.

Cancer has traditionally been viewed as a disease driven by the accumulation of genetic mutations. Lately, important roles for epigenetic abnormalities in cancer have come to light [26]. However, current cancer therapies still have limited effectiveness and serious side effects. Novel regimens are being tested, including immunotherapy targeting PD-1 and CTLA-4 [27],[28], oncolytic viruses [29], and HDAC inhibitors such as SAHA and MS-275 [30],[31]. Although these new therapies have proven therapeutic efficacy in a number of malignancies, better approaches are still urgently needed. MS-275 is currently in clinical trials for refractory and relapsed acute leukemia and advanced lymphoma [30]. Nevertheless, the application of HDAC inhibitors may be limited by the presence of HDAC mutations in certain cancers [32].

The clinical use of IFN-λ has a unique advantage over traditional IFN-α therapy, which is accompanied by toxicity due to the ubiquitously expressed IFN-α/β receptor. In addition to induction of apoptosis and cell cycle arrest by HDAC inhibitors [33], IFN-λ exhibits potent pro-apoptotic activity, thereby limiting the proliferation of HDAC inhibitor-resistant tumors. Furthermore, the combination is also superior to IFN-λ alone due to effectiveness in cancers previously insensitive to IFN-λ, including glioblastoma, which is known for its complexity and ability to develop resistance to single agent therapy [34]. This approach will need to be evaluated in a clinically relevant *in vivo* model system, and would be an important area for future study. In conclusion, our study elucidates the molecular mechanisms underlying the tissue specificity of IFN-λ receptor expression and provides a basis for the future development of antiviral and anticancer therapies.

## Materials and Methods

### Cells and Reagents

Huh7, HepG2, U87, U373, A549, 293T, BHK-21, B16, BNL, and NIH3T3 cells were maintained in high glucose DMEM supplemented with 10% heat-inactivated FBS, 100 U/ml penicillin, 100 µg/ml streptomycin, 2 mM L-glutamine, 1 mM sodium pyruvate, 1× MEM nonessential amino acids, and 10 mM HEPES (Invitrogen). Jurkat cells were cultured in complete RPMI 1640 medium. Cryopreserved PHHs from HIV, HBV, and HCV-free donors were purchased from BD Biosciences. Fresh PHHs were also obtained from the Cellular and Molecular Physiology core facility of the Yale Liver Center. Primary human astrocytes and HCMV-GFP were kindly provided by Dr. Anthony van den Pol (Yale University). Primary human T lymphocytes were originally obtained from the New York Blood Bank, and the CD4^+^ population was purified and kindly provided by Dr. Walther Mothes (Yale University). CD4^+^ T cells were cultured in complete RPMI 1640 medium supplemented with 100 U/ml IL-2 and 100 ng/ml IL-7 and stimulated with 10 µg/ml PHA every 72 h. Cells were treated with recombinant human IFN-α2a, IFN-β, human IFN-λ1, IFN-λ2, IFN-λ3 (R&D Systems), murine IFN-λ2 (PeproTech), or universal type I IFN (hIFN-αA/D) (PBL Interferon Source). DNMT inhibitor 5azadC and HDAC inhibitors SAHA, TSA, sodium butyrate, MS-275, apicidin, tubacin, and nicotinamide were purchased from Selleckchem.

### RNA Isolation, Reverse Transcription, and Real-Time Quantitative PCR (RT-qPCR)

Total RNA was harvested and extracted using RNeasy Mini Kit (Qiagen) according to the manufacturer's instructions. RNA was converted to cDNA using High Capacity cDNA Reverse Transcription Kit (Applied Biosystems). qPCR was performed using the 7500 Real Time PCR system (Applied Biosystems) with each reaction composed of cDNA reverse-transcribed from 50 ng of total RNA, 12.5 µl of Power SYBR Green master mix (Applied Biosystems), and 200 nM both forward and reverse primers in a total volume of 25 µl. For the detection of human IFNLR1 and IL10RB, the following TaqMan Gene Expression Assay primers and probe mixes were used instead of SYBR Green master mix: *IFNLR1* (Hs00417120_m1) and *IL10RB* (Hs00175123_m1). qPCR was performed using the following program: an initial incubation at 50°C for 2 min, a denaturation step at 95°C for 10 min, and 50 cycles consisting of 30 s at 95°C followed by 1 min at 60°C. Relative levels of gene expression were analyzed by ABI SDS v1.2.3 software (Applied Biosystems) with normalization to the constitutively expressed GAPDH using the ΔΔC_t_ method. The primers used for qPCR are listed in Table S1. The specificity of primers was verified by dissociation curve analysis, and the expected amplicon size of amplified products was visualized by 1.5% agarose gel electrophoresis.

### siRNA Transfection

Huh7 cells were seeded into 24-well plates and transfected with ON-TARGET plus SMARTpool siRNA against NF-YA (Thermo Scientific) or scrambled siRNA (Ambion) using DharmaFECT4 (Dharmacon). U87 cells were seeded into 24-well plates and transfected with FlexiTube siRNA against HDAC1 (Qiagen) or scrambled siRNA using DharmaFECT1. RNA was extracted from transfected cells 72 h later, and gene expression was determined by RT-qPCR.

### shRNA Transduction

HEK293T cells in six-well plates were transfected with pGIPZ lentiviral shRNA plasmid against NF-YA (Open Biosystems), Gag/Pol, and VSV-G packaging plasmids using Fugene 6 (Promega). Supernatants were harvested 48 h posttransfection and filtered through a 0.22 µm filter (Millipore). Huh7 or U373 cells were transduced with lentiviruses in the presence of polybrene (8 µg/ml). After 48 h, transduced cells were subject to puromycin selection (1 µg/ml) for 14 d. Stable NF-YA knockdown cells were maintained in low puromycin (400 ng/ml) medium.

### McrBC Cleavage Assay

A total of 1 µg of genomic DNA from Huh7 and U87 cells was digested with 10 units of *McrBC* (NEB) with or without 1 mM GTP in 50 µl reaction buffered with NEB buffer 2 at 37°C overnight. Digested DNA was purified with phenol/chloroform extraction followed by ethanol precipitation and subjected to PCR amplification using two pairs of nested PCR primers (see Table S1). PCR products were visualized by 1% agarose gel electrophoresis.

### Bisulfite Conversion Sequencing

Genomic DNA was extracted from Huh7, HepG2, U87, and U373 cells using phenol/chloroform extraction. Bisulfite conversion reactions were performed using EpiTect Bisulfite Kit (Qiagen) according to the manufacturer's instructions. The modified DNA was used as template for nested PCR with two sets of primers for both CpG islands in the *IFNLR1* promoter. Hot-start PCR was performed using GoTaq polymerase (Promega) at 95°C for 5 min, followed by a first-round amplification using 40 cycles of 95°C for 30 s, 45°C for 40 s, and 72°C for 1 min. The conditions for the second amplification were the same as those for the first round of PCR. The PCR products were electrophoresed in 2% agarose gels and visualized with ethidium bromide. Bands of expected sizes were excised, and the DNA was extracted using a Wizard SV Gel and PCR clean-up system (Promega). The purified DNA was cloned into pGEM-T vector (Promega). At least 20 clones were selected for each CpG island per cell type and sequenced by the Keck DNA Sequencing Facility (Yale University). The primers used in the nested PCR are listed in Table S1.

### ChIP

Huh7, U87 cells, and primary astrocytes were grown to 90% confluency on 10 cm cell culture dishes. After crosslinking at RT for 10 min with 1% formaldehyde, glycine was added to a final concentration of 0.125 M. The cells were washed three times with ice-cold PBS and scraped into PBS containing 1 mM PMSF. Cells were lysed with ChIP lysis buffer (140 mM NaCl, 1 mM EDTA, 0.25% Triton X-100, 10% glycerol, 0.5% NP-40 in 50 mM HEPES-KOH, pH 7.5) and subsequently with nuclei lysis buffer (100 mM NaCl, 1 mM EDTA, 0.5 mM EGTA, 0.1% sodium deoxycholate in 10 mM Tris-HCl, pH 8.0). The chromatin lysate was sonicated in 4°C water-bath using Bioruptor Standard for 15 min. Supernatants were collected from sonicated chromatin by centrifugation at 4°C at 13,200 rpm for 20 min. Before ChIP, chromatin was incubated with sheared salmon sperm DNA for pre-clearing. ChIP-grade antibodies against acetylated histone H3, RNA Polymerase II, H3K27me3, H3K9me3 (Millipore), and NF-YC (Santa Cruz) were incubated with precleared protein A/G magnetic beads (Pierce) at 4°C overnight. Antibody/beads complexes were mixed with precleared chromatin at 4°C overnight. ChIP complexes were washed in order with high-salt, low-salt, LiCl wash buffers, and TE water. Bound DNA was released by reversing the crosslinking and subject to qPCR analysis. The primers used for qPCR reactions are listed in Table S1.

### EMSA

Gel shift assays were performed using radiolabeled EMSA probes with dialyzed nuclear extracts from U87 cells. Cells grown to 90% confluency from 10 cm cell culture dishes were harvested in hypotonic buffer (10 mM KCl, 10 mM HEPES, 1.5 mM MgCl_2_, 0.5 mM DTT, 1 mM PMSF, 5 µg/ml leupeptin), homogenized with a type B Dounce homogenizer, and further lysed in nuclear lysis buffer (0.42 M NaCl, 20 mM HEPES, 1.5 mM MgCl_2_, 0.2 mM EDTA, 25% glycerol, 1 mM DTT, 2 mM PMSF, 5 µg/ml leupeptin). Nuclear extracts were dialyzed against 1× binding buffer (20 mM HEPES, 50 mM KCl, 1 mM β-ME, 10% glycerol, 0.5 mM DTT, 1 mM PMSF) at 4°C overnight. EMSA probes were generated by annealing two strands of oligonucleotides from sequences in the *IFNLR1* promoter. Probes were end-labeled with α-^32^P-dCTP using Klenow reaction from Random Primed DNA Labeling Kit (Roche). For each EMSA reaction, 50,000 cpm probe was incubated with 10 µl of 2× binding buffer, 1 µl of poly (dA∶dT), 5 µg of nuclear protein in a 20 µl reaction with or without 100-fold excess of cold competitor probe, and antibodies against NF-YA, NF-YB, NF-YC, E2F1, and E2F4. Protein–DNA complexes were separated by 6% native polyacrylamide gels in low-ionic strength buffer system. Gels were dried at 80°C for 1 h and exposed to Maximum Resolution film (Kodak) at −80°C for 3 d. The sequences of oligonucleotides used in this study are listed in Table S1.

### *In Vitro* Methylation

EMSA probe was methylated *in vitro* with methyltransferase *M.SssI* (NEB) and S-adenosylmethionine (SAM) as the methyl donor. A total of 5 µg of probe was incubated with 40 units of *M.SssI* and 1 µl of SAM (32 mM stock concentration) in 150 µl reaction buffered with NEB buffer 2 at 37°C overnight. The next day the reaction was supplemented with an additional 1 µl of SAM and further incubated for 2 h. Methylated probe was isolated by phenol/chloroform extraction and ethanol precipitation. Complete CpG methylation was verified by digestion with the methylation-sensitive restriction enzyme *HpaII* (NEB).

### Histone Extraction

U87 cells were treated with HDAC inhibitors, harvested in high salt lysis buffer (320 mM NaCl, 0.1 mM EDTA, 0.5% NP-40 in 50 mM Tris-HCl, pH 7.6), and incubated on ice for 10 min. After concentrifugation at 13,200 rpm for 10 min at 4°C, supernatants were lysed in 1× SDS loading buffer (2% SDS, 100 mM DTT, 0.1% bromophenol blue, 10% glycerol in 50 mM Tris-HCl, pH 6.8). Lysates were sonicated for 15 min, heated at 95°C for 10 min, and used in Western blotting (WB).

### WB

U87 cells were washed with ice-cold PBS before lysis in RIPA buffer (150 mM sodium chloride, 1.0% Triton X-100, 0.5% sodium deoxycholate, 0.1% SDS, 50 mM Tris, pH 8.0). Cells were scraped off from six-well plates and agitated at 4°C for 10 min. After centrifugation at 12,000 rpm at 4°C for 20 min, supernatants were mixed at 1∶1 ratio with 2× Laemmli buffer. Protein lysates were heated at 95°C for 5 min, separated by 10% Mini-PROTEAN gels (Bio-Rad), transferred onto 0.45 µm nitrocellulose membranes, and probed with antibodies against p-STAT1 (Y701), total STAT1, NF-κB p65, p-Akt, total Akt, p-mTOR, total mTOR, PKR (Cell Signaling), VSV, GFP, NF-YA, NF-YB, NF-YC, p-p70 S6 kinase, total p70 S6 kinase, PI3K, p21, Rb, caspase-3, caspase-9, XIAP, survivin, β-tubulin (Santa Cruz), acetylated H3 (Millipore), and total histone H3 (Abcam). Secondary incubation was performed with anti-goat, anti-rabbit, or anti-mouse IgG HRP-linked antibodies (Cell Signaling). Proteins were visualized using HyGlo chemiluminescent HRP antibody detection reagent (Denville Scientific Inc.) and a Fuji LAS-3000 cooled CCD camera (FUJIFILM).

### CPE Assay

U87 cells in 24-well plates were treated with DMSO or 5azadC plus MS-275 as previously described, stimulated with 10, 30, or 100 ng/ml IFN-λ or 500 U/ml IFN-β for 24 h, and infected with wild-type VSV at MOI = 1. At 24 h postinfection, cells were fixed with 2% paraformaldehyde (PFA) at RT for 10 min, washed with PBS, and stained with crystal violet (0.5% in 25% methanol) at RT for 10 min.

### Plaque Assay

Drug-treated U87 cells were stimulated with IFN-λ or IFN-β before VSV infection. Supernatants were harvested 24 h postinfection and diluted to 10, 10^2^, 10^3^, 10^4^, 10^5^, 10^6^, and 10^7^ fold. Diluted viruses were transferred onto BHK-21 cells for a 30 min infection at 37°C. Viruses were then removed and replaced by 1% methylcellulose. Virus titers were determined 2 d later by quantification of GFP-positive cells.

### Cell Proliferation/Viability Assay

Inhibitor-treated cells in 96-well plates were incubated with 100 µl of complete medium containing 10 µl of WST-1 (Roche) and incubated at 37°C for 0.5 h. Absorbance was measured at 440 nm by the basic endpoint protocol using a SpectraMAX 190 microplate reader (Molecular Devices). Wells containing reagent alone were used as blank control for background subtraction. Data were analyzed by SoftMax Pro 4.7.1 (Molecular Devices).

### Three-Dimensional Spheroid Culture

AlgiMatrix 3D Culture System (Invitrogen) was used for constituting artificial bioscaffold to facilitate the formation of U87 spheroids in three dimensions. U87 cells were seeded into AlgiMatrix six-well plates and grown to form spheroids for 2 wk following the manufacturer's instructions. After MS-275 or IFN-λ treatment, images were acquired in the bright field and cells were harvested for WST-1 assay. CellProfiler 2.0 software (http://www.cellprofiler.org/) was used for image processing to detect and quantify individual spheroids.

### Flow Cytometry

MS-275 or IFN-λ-treated U87 cells in six-well plates were trypsinized and stained with propidium iodide (Fisher) or Annexin V-FITC plus 7-AAD (BioLegend). Stained cells were washed with PBS, and fluorescence was measured with a 13-color Stratedigm S1000Ex flow cytometry platform. Laser light scatter was used to gate out dead or dying cells. For HSV-1 and VSV experiments, infected astrocytes were fixed with PFA, stained for live/dead cells (BioLegend), and examined using the Stratedigm flow cytometer for GFP expression. Data were analyzed with FlowJo Software v8.8.7 (TreeStar).

### Senescence-Associated β-Galactosidase Activity Assay

MS-275 or IFN-λ-treated U87 cells in six-well plates were fixed with 1 ml of 0.2% glutaraldehyde for 15 min at RT, washed twice with PBS, and incubated with staining solution (5 mM potassium ferrocyanide, 5 mM potassium ferricyanide, 2 mM MgCl_2_, 1 mg/ml X-gal). Cells were incubated in dark at 37°C for 16 h. Images at 200× magnification were acquired and analyzed with CellProfiler 2.0.

### HSV-1 Infection

Primary astrocytes were cultured in the presence of DMSO or 5azadC and MS-275 as previously described, stimulated with 100 ng/ml IFN-λ or 500 U/ml IFN-β for 24 h, and infected with HSV-1-GFP at MOI = 1 at 37°C for 1 h. Cells were harvested 12 h postinfection for flow cytometry or RT-qPCR analysis.

### Statistical Analysis

The results are shown as means ± SEM. Statistical significance was determined by Student's *t* test using Prism 6 (GraphPad Software). Significant differences are indicated on figures (*p≤0.05; **p≤0.01; ***p≤0.001).

## Supporting Information

Figure S1 **Two putative CpG islands are located in close proximity to the TSS in the** ***IFNLR1*** **gene locus.** (A) CpG islands in the *IFNLR1* promoter were identified with EMBOSS Cpgplot (European Bioinformatics Institute) using the default settings. Lower numbers indicate relative distance to the TSS. (B) Genomic DNA was isolated from PHHs and used for bisulfite conversion sequencing. Each circle represents one CpG dinucleotide, with filled circles indicating methylated motifs and open circles nonmethylated motifs. Each row represents an individual clone of the population. Lower numbers indicate relative distance to the TSS. (C) Quantification of the methylation status of both CpG islands in (B). (D) U87 cells were cultured in the presence of 3 µM 5azadC for 72 h. Genomic DNA was isolated and used for bisulfite conversion sequencing. (E) Quantification of the methylation status on CpG island II in (D).
(TIF)

Figure S2 **DNMT and HDAC inhibitors up-regulate IFN-λ receptor expression in a wide range of nonresponsive cell types.** (A) ChIP analysis was performed on the *IFNLR1* promoter in Huh7 and U87 cells with H3K9me3 and control IgG antibodies. (B) ChIP analysis was performed on the *IFNLR1* promoter in primary astrocytes with AcH3 and control IgG antibodies. (C) Lysates of inhibitor-treated U87 cells were used for WB using indicated antibodies. (D) U87 cells were cultured with DMSO or 10 µM 5azadC for 72 h. For the latter, 1 µM Trichostatin A (TSA), 10 mM sodium butyrate (NaBu), 5 mM nicotinamide (NAM), or 0.5 µM apicidin were added in the last 24 h. IFNLR1 expression was determined by RT-qPCR. (E–F) U87 cells were cultured in the presence of DMSO or 5azadC with/without MS-275, and transfected with scrambled or HDAC1-specific siRNAs. HDAC1 and IFNLR1 expression was examined by RT-qPCR. (G–L) Huh7 (human liver hepatoma), A549 (human lung adenocarcinoma), Jurkat (human T lymphoma), BNL (mouse hepatocellular carcinoma), NIH3T3 (mouse embryonic fibroblast), and primary human CD4^+^ T cells were cultured in the presence of DMSO or 10 µM 5azadC for 72 h. A total of 1 µM MS-275 was added to 5azadC-treated cells in the last 24 h. IFNLR1 expression was determined by RT-qPCR. In all panels, data represent the mean and SEM of at least three experiments.
(TIF)

Figure S3 **The −434 to −401 region of the** ***IFNLR1*** **promoter interacts with** ***trans*** **-acting factors.** (A) Bioinformatics prediction of TF binding sites was performed using the TRANSFAC v10.2 database. TFs with potential binding capacity were annotated with putative binding matrix indicated as stretches of colored bars. Box highlights NF-Y and E2F family proteins. Lower numbers indicate relative distance to the TSS. (B) Gel mobility shift assay was performed using three ^32^P-radiolabeled DNA probes that covered the −500∼−401 region of the *IFNLR1* promoter, incubated with U87 cell nuclear extracts, poly (dA∶dT), and excessive cold competitor probe. (C) Gel mobility shift assay was performed using the −434∼−401 mutant probes in which adjacent five nucleotides were converted to consecutive adenines, as is illustrated with red crosses on solid black lines (see detailed sequence information in Table S1).
(TIF)

Figure S4 **NF-Y is ubiquitously expressed, and its knockdown in nonresponsive cells does not affect IFN-λ receptor expression.** (A–B) Gel mobility shift assay was performed using the wild-type (WT) probe (the −434∼−401 region of the *IFNLR1* promoter) and the methylated (ME) probe (WT probe after *M.SssI* treatment), which were incubated with poly (dA∶dT), U87 cell nuclear extracts, excessive cold competitor probe, and indicated antibodies. (C–D) Expression of NF-YA, NF-YB, and NF-YC in different cell types was measured by RT-qPCR and WB with indicated antibodies. (E–F) NF-YA and IFNLR1 expression was determined by RT-qPCR in U87 cells transfected with scrambled or NF-YA-specific siRNAs. (G–H) NF-YA and IFNLR1 expression was measured by RT-qPCR in U373 cells stably expressing scrambled or NF-YA-specific shRNAs. In all panels, data represent the mean and SEM of at least three experiments.
(TIF)

Figure S5 **Small-molecule inhibitors increase IFN-λ sensitivity in U87 cells without affecting NF-Y expression.** (A) Expression of NF-YA, NF-YB, and NF-YC was determined by RT-qPCR in U87 cells post-DNMT and -HDAC inhibitor treatment. (B) Lysates from U87 cells with indicated treatment were used for WB using indicated antibodies. (C–E) U87 cells were treated with or without 5azadC and MS-275, and stimulated in the presence or absence of 100 ng/ml IFN-λ1 for 24 h. Expression of representative ISGs, such as IFI27 (P27), CXCL10 (IP-10), and ISG15 (G1P2), was determined by RT-qPCR. (F) Primary astrocytes were preincubated with DMSO or small-molecule inhibitors and stimulated with or without 100 ng/ml of IFN-λ1 for 6 h. Lysates were used for WB using the indicated antibodies. In all panels, data represent the mean and SEM of at least three experiments.
(TIF)

Figure S6 **Inhibitor-primed astrocytes are protected from VSV infection by IFN-λ.** (A–C) Primary astrocytes were treated with or without 5azadC and MS-275, stimulated with 100 ng/ml IFN-λ or 500 U/ml IFN-β for 24 h, and infected with VSV-GFP at MOI = 1, 0.1, or 0.01. At 24 h postinfection, cells were harvested, fixed, and examined for GFP expression by flow cytometry.
(TIF)

Figure S7 **Inhibitor-primed astrocytes are protected from HCMV infection by IFN-λ.** (A–C) Primary astrocytes were treated with or without 5azadC and MS-275, stimulated with 100 ng/ml IFN-λ or 500 U/ml IFN-β for 24 h, and infected with HCMV-GFP at MOI = 10, 1, or 0.1. At 48 h postinfection, GFP expression was determined by RT-qPCR.
(TIF)

Figure S8 **Suppression of tumor growth is independent of cellular senescence and mediated via apoptotic pathways.** (A, F) Cell proliferation of primary human astrocytes or mouse B16 melanoma cells with indicated treatment were performed using WST-1 assay, which measures active cellular metabolism. (B) U87 cells were cultured in the presence of DMSO, 1 µM MS-275, 100 ng/ml IFN-λ1, or both for 4 d. Cytochemical staining for SA-β-gal activity was monitored by inverted microscopy (representative images are shown; 200× magnification). (C) Percentage of SA-β-gal positive cells in (B) was quantified with CellProfiler (Broad Institute). (D) Lysates from U87 cells with indicated treatment were used for WB using indicated antibodies. (E) B16 cells were treated with 10 ng/ml murine IFN-λ2 or 10 U/ml universal type I IFN for 24 h and MX1 expression was measured by RT-qPCR. In all panels, data represent the mean and SEM of at least three experiments.
(TIF)

Table S1 **Oligonucleotides used for PCR, RT-qPCR, and EMSA experiments.** For EMSA probes, underlining indicates sites of mutation.
(DOCX)

## Abbreviations

CNS: central nervous system
DNMT: DNA methyltransferase
HAT: histone acetyltransferase
HCMV: human cytomegalovirus
HDAC: histone deacetylase
HSV: herpes simplex virus
IFN: interferon
ISG: interferon-stimulated gene
PHH: primary human hepatocyte
TF: transcription factor
VSV: vesicular stomatitis virus
