# Supplementary figures and images for: Epigenetic Reprogramming of the Type III Interferon Response Potentiates Antiviral Activity and Suppresses Tumor Growth
Source: PLoS Biol. 2014 Jan 7;12(1):e1001758. doi: 10.1371/journal.pbio.1001758 (PMC3883642; doi:10.1371/journal.pbio.1001758)

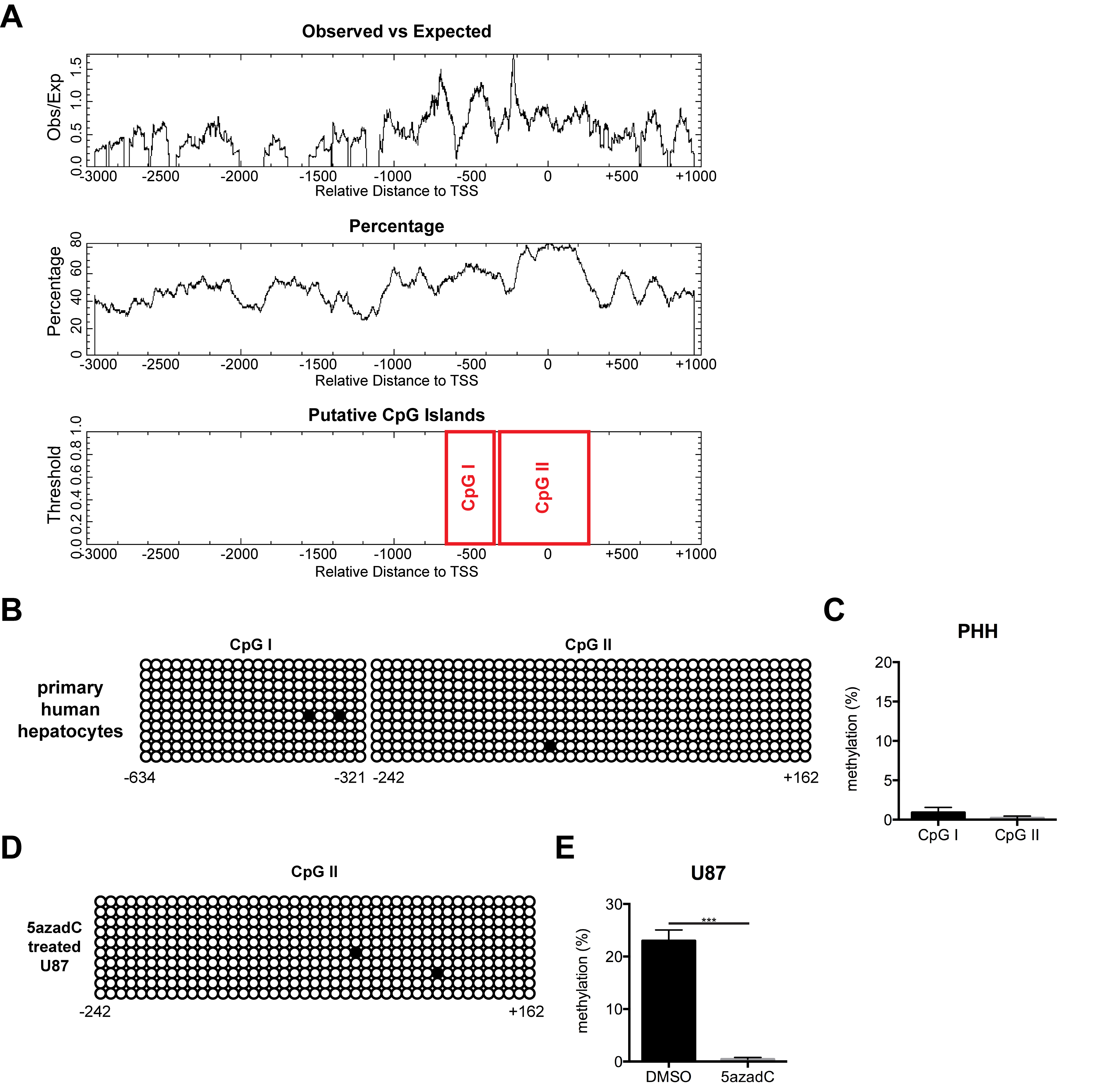

Supplement: Figure S1 — Two putative CpG islands are located in close proximity to the TSS in the IFNLR1 gene locus. (A) CpG islands in the IFNLR1 promoter were identified with EMBOSS Cpgplot (European Bioinformatics Institute) using the default settings. Lower numbers indicate relative distance to the TSS. (B) Genomic DNA was isolated from PHHs and used for bisulfite conversion sequencing. Each circle represents one CpG dinucleotide, with filled circles indicating methylated motifs and open circles nonmethylated motifs. Each row represents an individual clone of the population. Lower numbers indicate relative distance to the TSS. (C) Quantification of the methylation status of both CpG islands in (B). (D) U87 cells were cultured in the presence of 3 µM 5azadC for 72 h. Genomic DNA was isolated and used for bisulfite conversion sequencing. (E) Quantification of the methylation status on CpG island II in (D). (TIF) [file pbio.1001758.s001.tif]

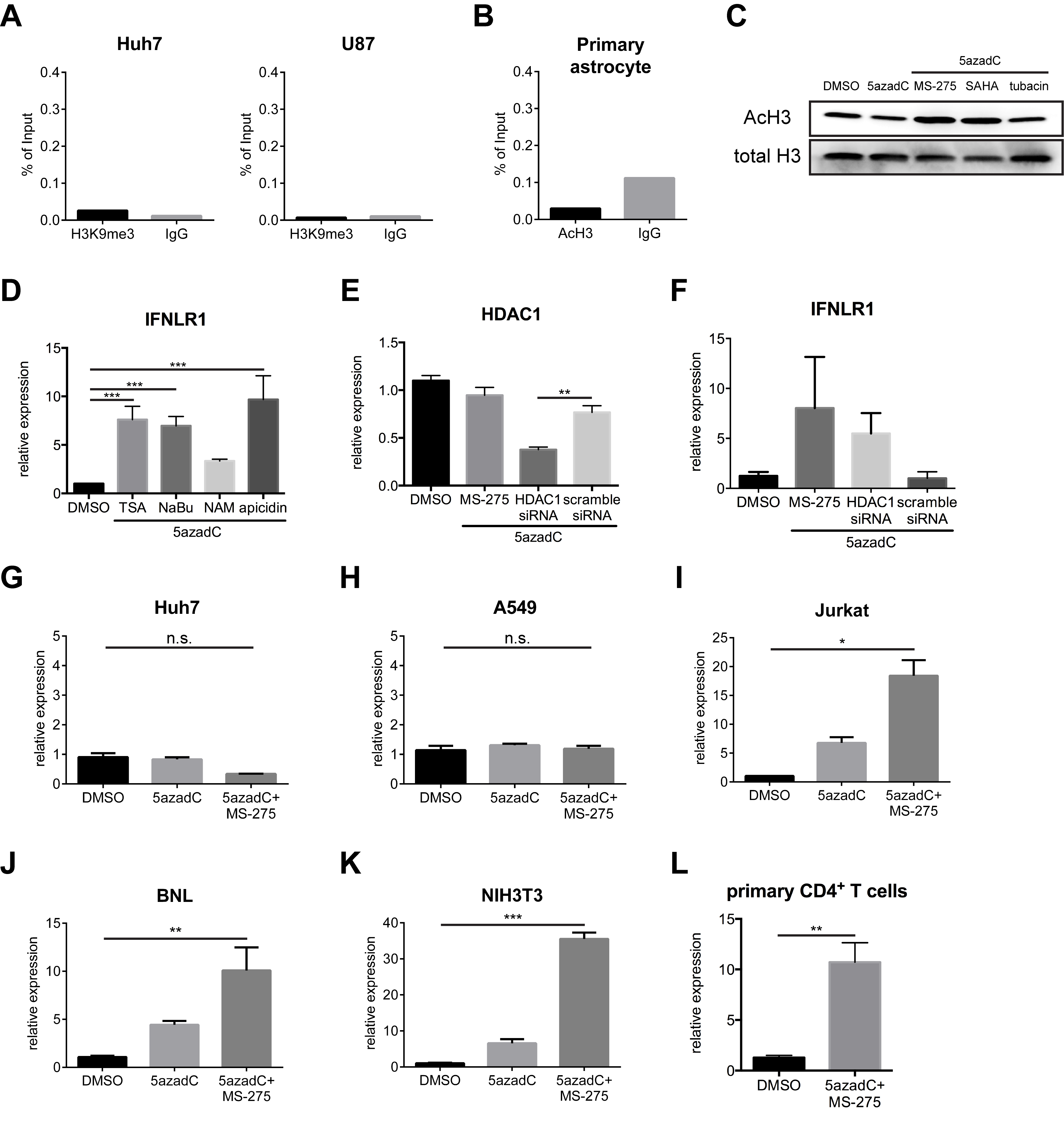

Supplement: Figure S2 — DNMT and HDAC inhibitors up-regulate IFN-λ receptor expression in a wide range of nonresponsive cell types. (A) ChIP analysis was performed on the IFNLR1 promoter in Huh7 and U87 cells with H3K9me3 and control IgG antibodies. (B) ChIP analysis was performed on the IFNLR1 promoter in primary astrocytes with AcH3 and control IgG antibodies. (C) Lysates of inhibitor-treated U87 cells were used for WB using indicated antibodies. (D) U87 cells were cultured with DMSO or 10 µM 5azadC for 72 h. For the latter, 1 µM Trichostatin A (TSA), 10 mM sodium butyrate (NaBu), 5 mM nicotinamide (NAM), or 0.5 µM apicidin were added in the last 24 h. IFNLR1 expression was determined by RT-qPCR. (E–F) U87 cells were cultured in the presence of DMSO or 5azadC with/without MS-275, and transfected with scrambled or HDAC1-specific siRNAs. HDAC1 and IFNLR1 expression was examined by RT-qPCR. (G–L) Huh7 (human liver hepatoma), A549 (human lung adenocarcinoma), Jurkat (human T lymphoma), BNL (mouse hepatocellular carcinoma), NIH3T3 (mouse embryonic fibroblast), and primary human CD4+ T cells were cultured in the presence of DMSO or 10 µM 5azadC for 72 h. A total of 1 µM MS-275 was added to 5azadC-treated cells in the last 24 h. IFNLR1 expression was determined by RT-qPCR. In all panels, data represent the mean and SEM of at least three experiments. (TIF) [file pbio.1001758.s002.tif]

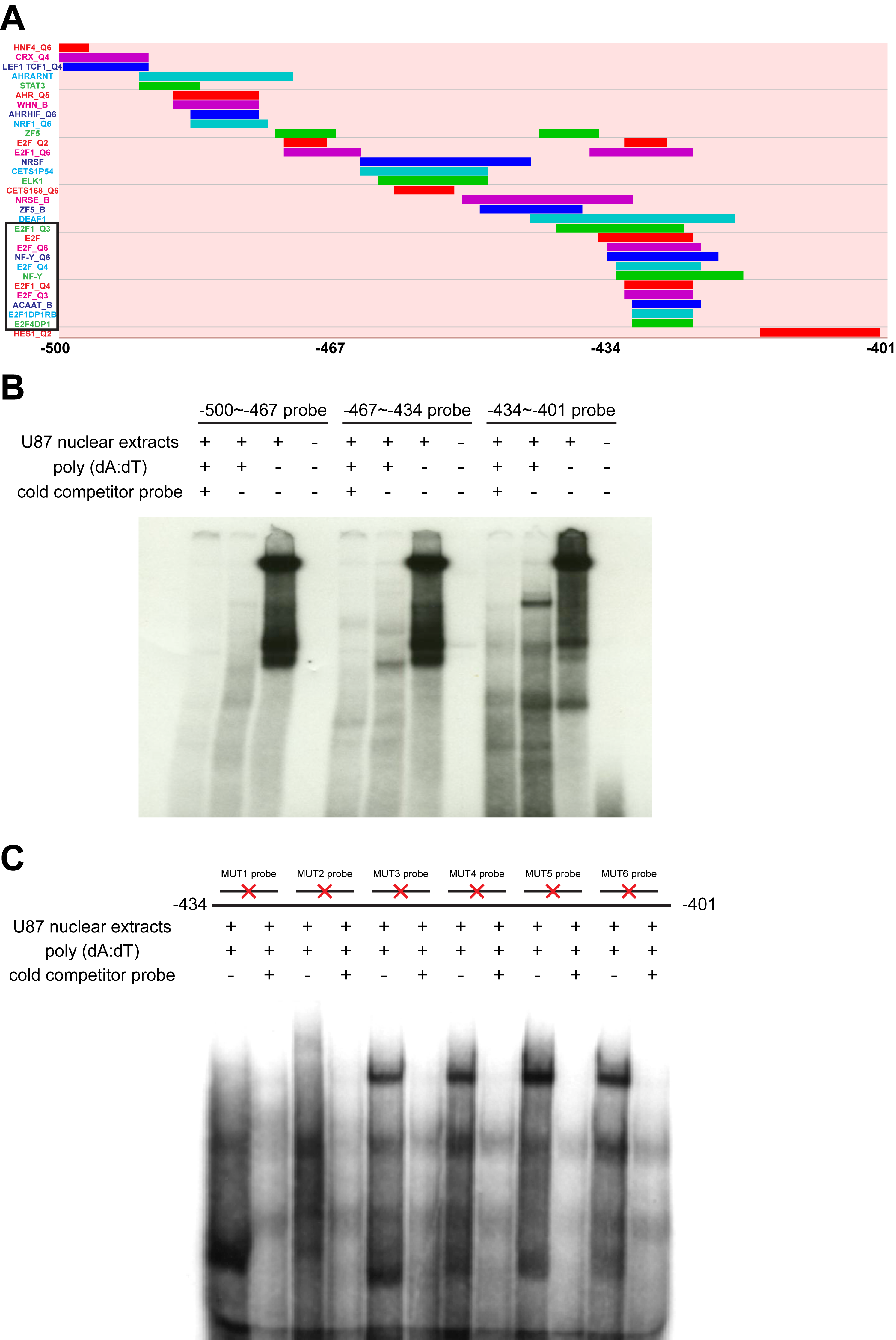

Supplement: Figure S3 — The −434 to −401 region of the IFNLR1 promoter interacts with trans -acting factors. (A) Bioinformatics prediction of TF binding sites was performed using the TRANSFAC v10.2 database. TFs with potential binding capacity were annotated with putative binding matrix indicated as stretches of colored bars. Box highlights NF-Y and E2F family proteins. Lower numbers indicate relative distance to the TSS. (B) Gel mobility shift assay was performed using three 32P-radiolabeled DNA probes that covered the −500∼−401 region of the IFNLR1 promoter, incubated with U87 cell nuclear extracts, poly (dA∶dT), and excessive cold competitor probe. (C) Gel mobility shift assay was performed using the −434∼−401 mutant probes in which adjacent five nucleotides were converted to consecutive adenines, as is illustrated with red crosses on solid black lines (see detailed sequence information in Table S1). (TIF) [file pbio.1001758.s003.tif]

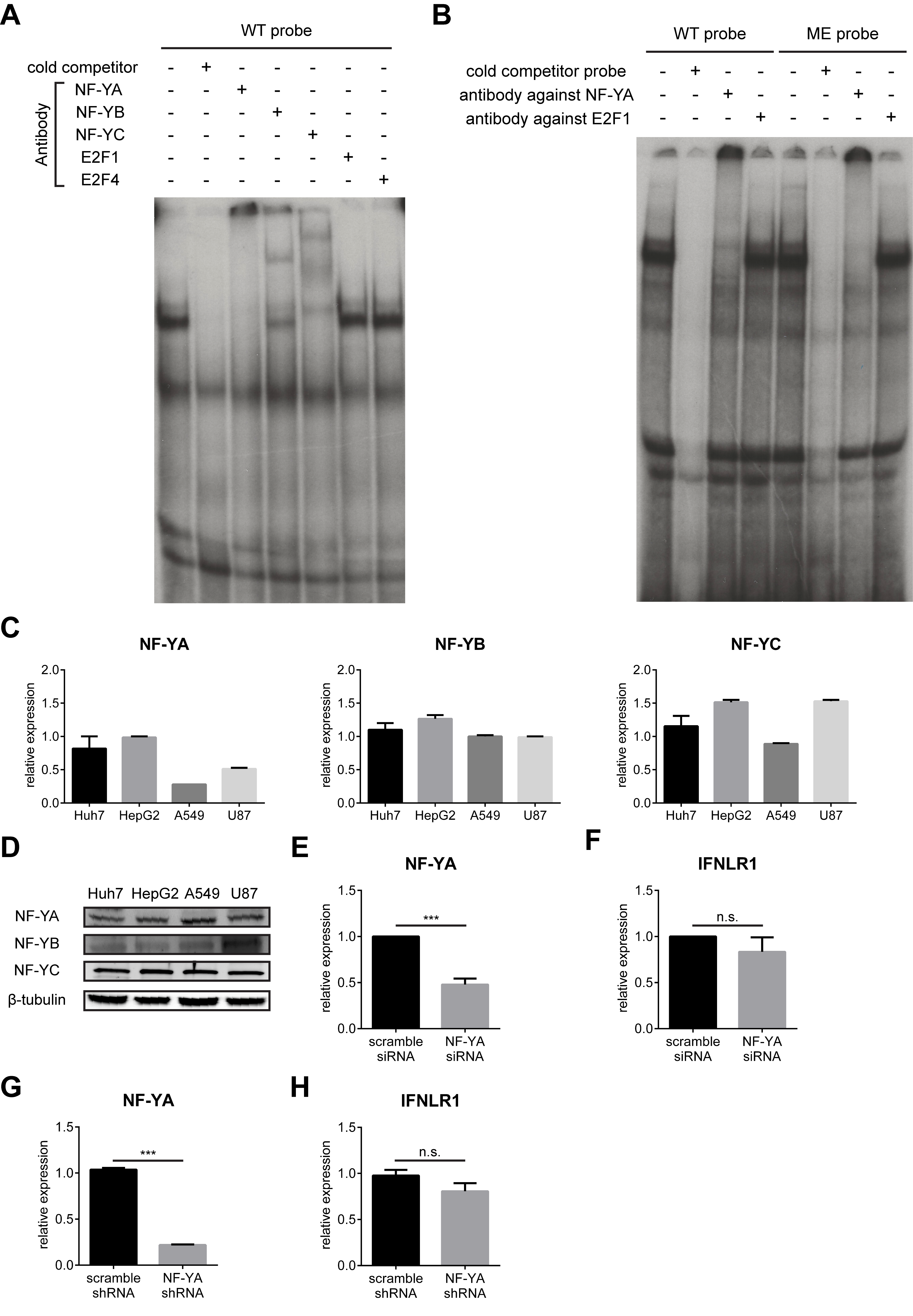

Supplement: Figure S4 — NF-Y is ubiquitously expressed, and its knockdown in nonresponsive cells does not affect IFN-λ receptor expression. (A–B) Gel mobility shift assay was performed using the wild-type (WT) probe (the −434∼−401 region of the IFNLR1 promoter) and the methylated (ME) probe (WT probe after M.SssI treatment), which were incubated with poly (dA∶dT), U87 cell nuclear extracts, excessive cold competitor probe, and indicated antibodies. (C–D) Expression of NF-YA, NF-YB, and NF-YC in different cell types was measured by RT-qPCR and WB with indicated antibodies. (E–F) NF-YA and IFNLR1 expression was determined by RT-qPCR in U87 cells transfected with scrambled or NF-YA-specific siRNAs. (G–H) NF-YA and IFNLR1 expression was measured by RT-qPCR in U373 cells stably expressing scrambled or NF-YA-specific shRNAs. In all panels, data represent the mean and SEM of at least three experiments. (TIF) [file pbio.1001758.s004.tif]

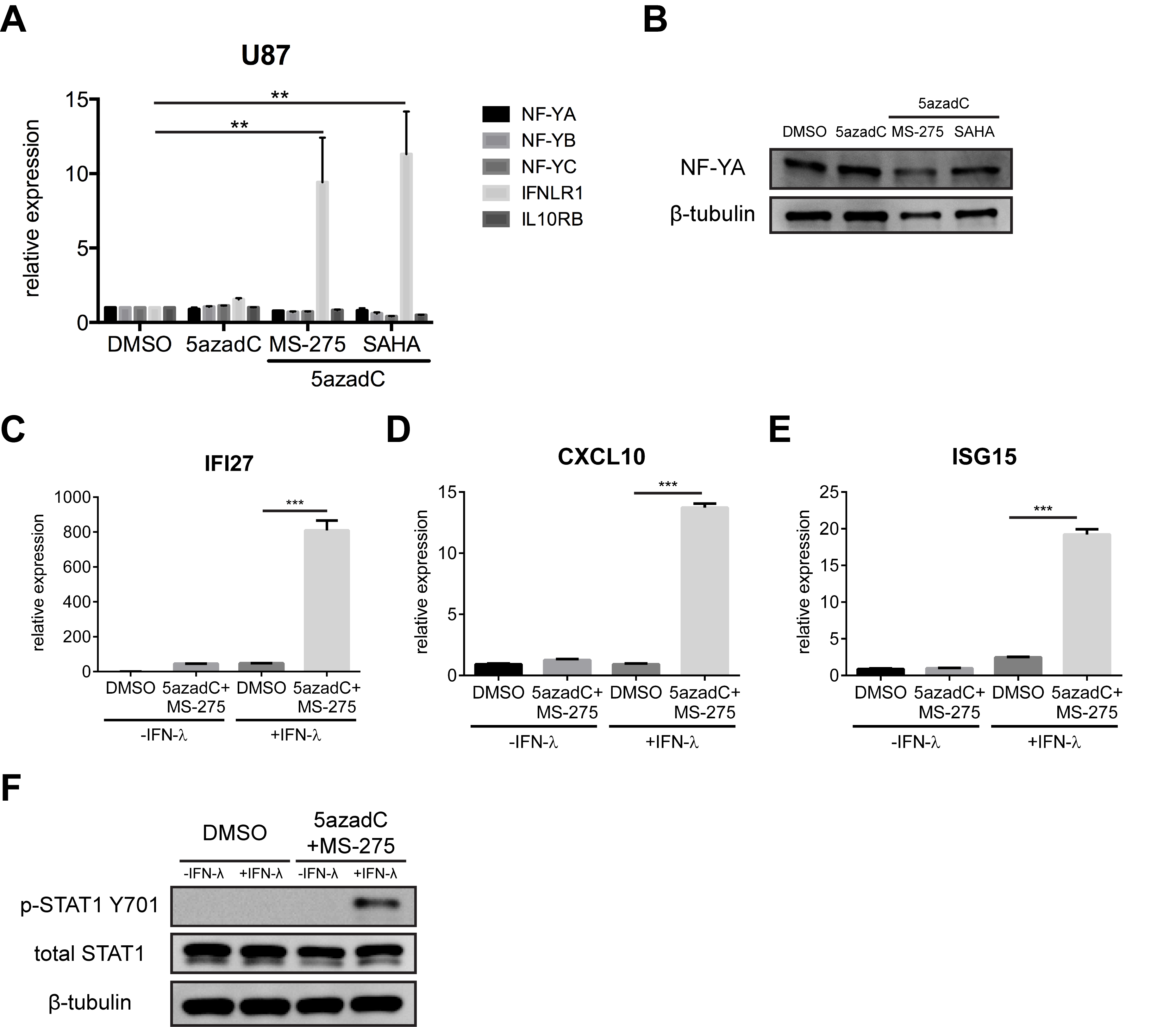

Supplement: Figure S5 — Small-molecule inhibitors increase IFN-λ sensitivity in U87 cells without affecting NF-Y expression. (A) Expression of NF-YA, NF-YB, and NF-YC was determined by RT-qPCR in U87 cells post-DNMT and -HDAC inhibitor treatment. (B) Lysates from U87 cells with indicated treatment were used for WB using indicated antibodies. (C–E) U87 cells were treated with or without 5azadC and MS-275, and stimulated in the presence or absence of 100 ng/ml IFN-λ1 for 24 h. Expression of representative ISGs, such as IFI27 (P27), CXCL10 (IP-10), and ISG15 (G1P2), was determined by RT-qPCR. (F) Primary astrocytes were preincubated with DMSO or small-molecule inhibitors and stimulated with or without 100 ng/ml of IFN-λ1 for 6 h. Lysates were used for WB using the indicated antibodies. In all panels, data represent the mean and SEM of at least three experiments. (TIF) [file pbio.1001758.s005.tif]

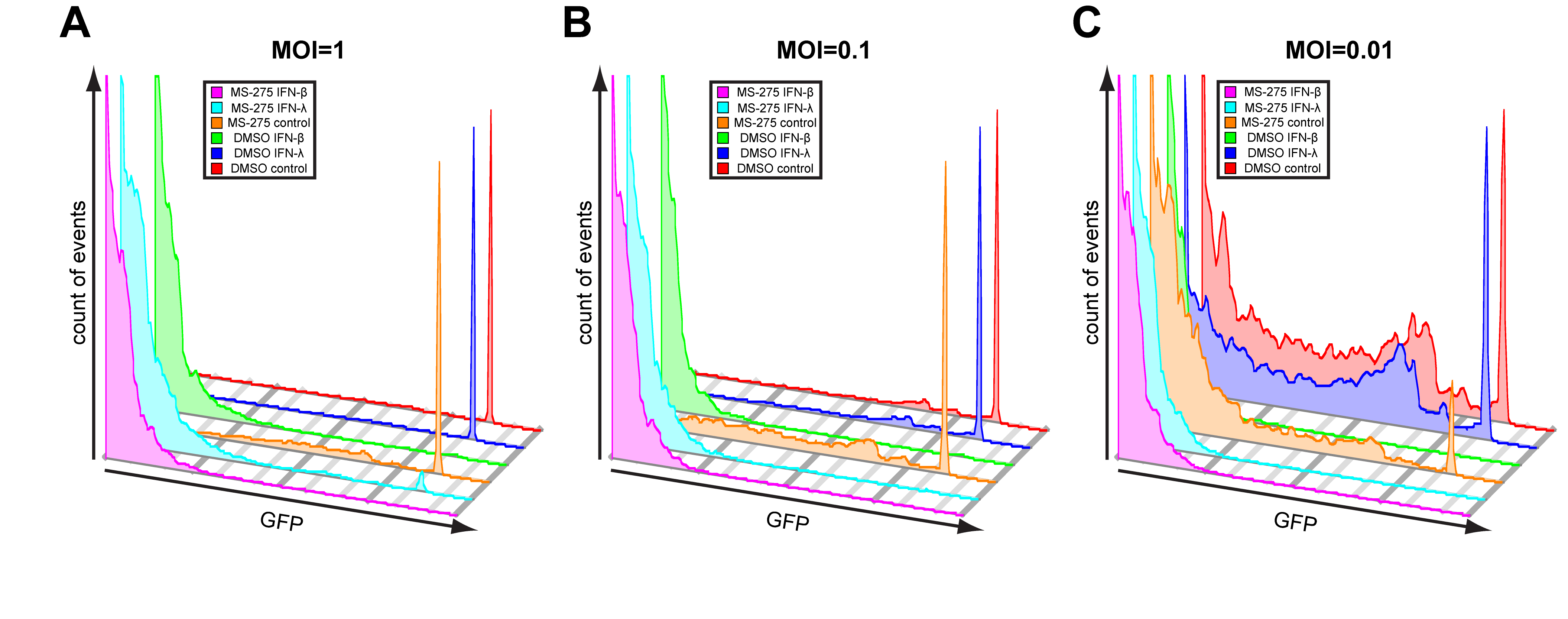

Supplement: Figure S6 — Inhibitor-primed astrocytes are protected from VSV infection by IFN-λ. (A–C) Primary astrocytes were treated with or without 5azadC and MS-275, stimulated with 100 ng/ml IFN-λ or 500 U/ml IFN-β for 24 h, and infected with VSV-GFP at MOI = 1, 0.1, or 0.01. At 24 h postinfection, cells were harvested, fixed, and examined for GFP expression by flow cytometry. (TIF) [file pbio.1001758.s006.tif]

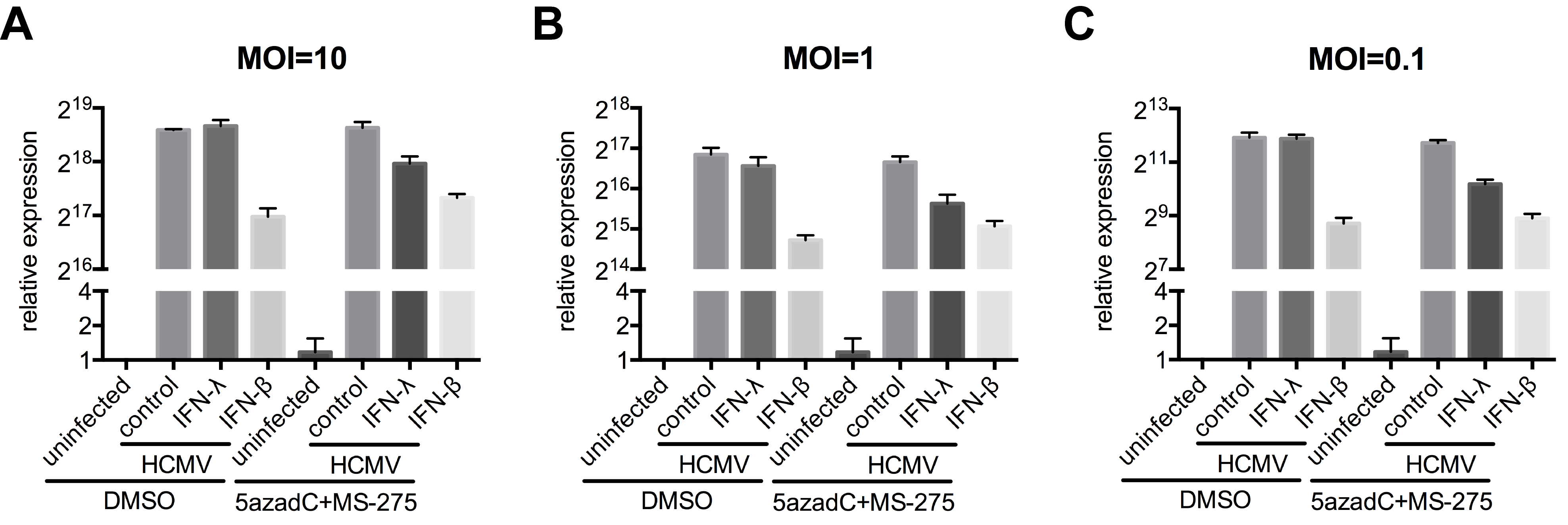

Supplement: Figure S7 — Inhibitor-primed astrocytes are protected from HCMV infection by IFN-λ. (A–C) Primary astrocytes were treated with or without 5azadC and MS-275, stimulated with 100 ng/ml IFN-λ or 500 U/ml IFN-β for 24 h, and infected with HCMV-GFP at MOI = 10, 1, or 0.1. At 48 h postinfection, GFP expression was determined by RT-qPCR. (TIF) [file pbio.1001758.s007.tif]

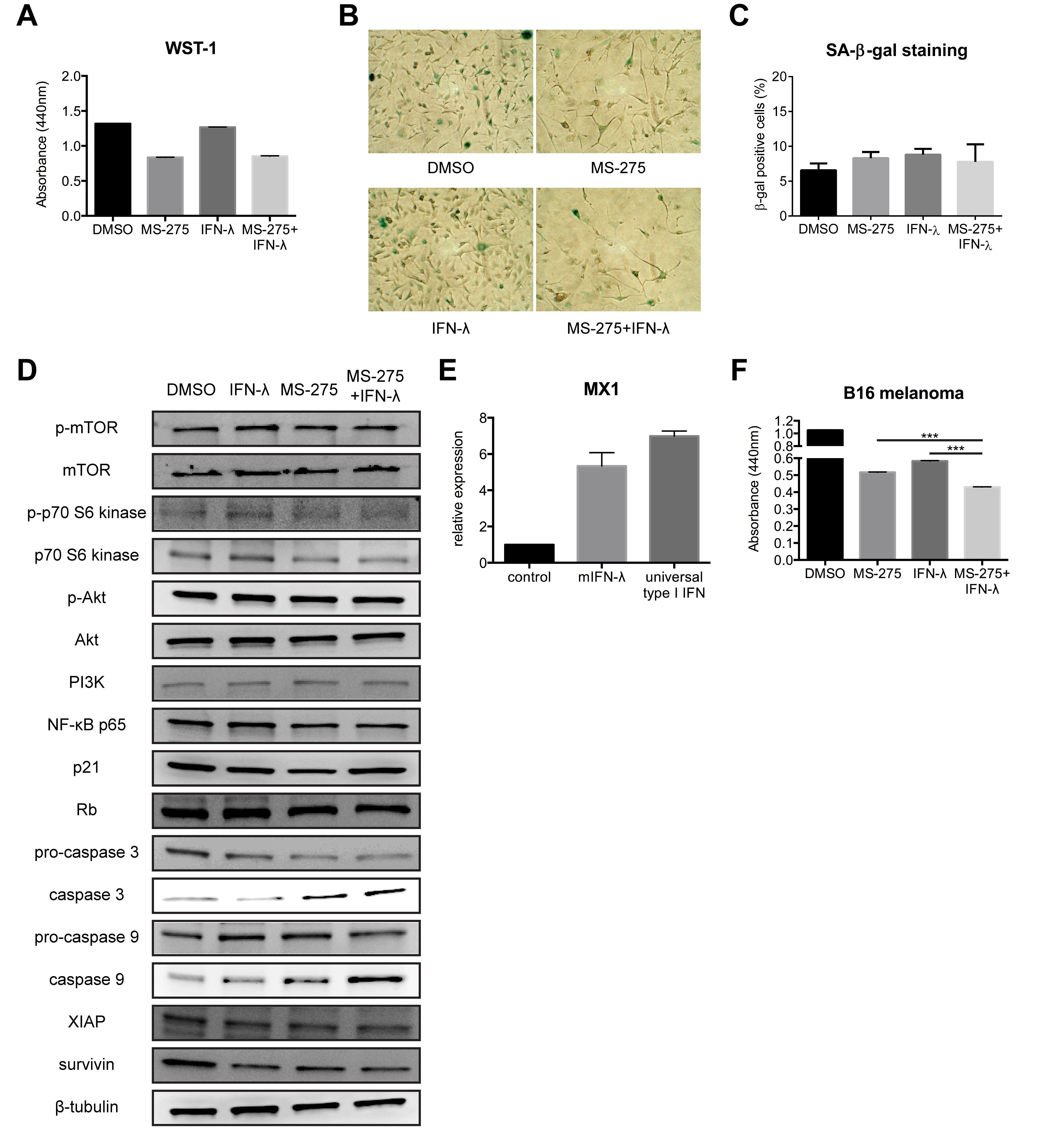

Supplement: Figure S8 — Suppression of tumor growth is independent of cellular senescence and mediated via apoptotic pathways. (A, F) Cell proliferation of primary human astrocytes or mouse B16 melanoma cells with indicated treatment were performed using WST-1 assay, which measures active cellular metabolism. (B) U87 cells were cultured in the presence of DMSO, 1 µM MS-275, 100 ng/ml IFN-λ1, or both for 4 d. Cytochemical staining for SA-β-gal activity was monitored by inverted microscopy (representative images are shown; 200× magnification). (C) Percentage of SA-β-gal positive cells in (B) was quantified with CellProfiler (Broad Institute). (D) Lysates from U87 cells with indicated treatment were used for WB using indicated antibodies. (E) B16 cells were treated with 10 ng/ml murine IFN-λ2 or 10 U/ml universal type I IFN for 24 h and MX1 expression was measured by RT-qPCR. In all panels, data represent the mean and SEM of at least three experiments. (TIF) [file pbio.1001758.s008.tif]
