# Supplementary material for: Epigenetic Reprogramming of the Type III Interferon Response Potentiates Antiviral Activity and Suppresses Tumor Growth
Source: PLoS Biol. 2014 Jan 7;12(1):e1001758. doi: 10.1371/journal.pbio.1001758 (PMC3883642; doi:10.1371/journal.pbio.1001758)
Supplement: Table S1 — Oligonucleotides used for PCR, RT-qPCR, and EMSA experiments. For EMSA probes, underlining indicates sites of mutation. (DOCX) [file pbio.1001758.s009.docx]

**Table S1. Oligonucleotides used for PCR and EMSA**

| **SYBR Green qPCR primers** | Forward | | Reverse | |
| --- | --- | --- | --- | --- |
| NF-YA | 5'CAGTGGAGGCCAGCTAATCAC | | 5'CCAGGTGGGACCAACTGTATT | |
| NF-YB | 5'AGCAAACGTGGCTAGGATAATG | | 5'AGGTTCCACATAACTGTCAAAGC | |
| NF-YC | 5'GGAGGATTTGGTGGTACTAGCA | | 5'GCACTCGGAAGTCTTTCACTG | |
| MX1 | 5'GTGGCTGAGAACAACCTGTG | | 5'GGCATCTGGTCACGATCCC | |
| IFI27 | 5'TGCTCTCACCTCATCAGCAGT | | 5'CACAACTCCTCCAATCACAACT | |
| CXCL10 | 5'GTGGCATTCAAGGAGTACCTC | | 5'TGATGGCCTTCGATTCTGGATT | |
| ISG15 | 5'AATGCGACGAACCTCTGAGC | | 5'CAGCCAGAACAGGTCGTC | |
| GAPDH | 5'CATGAGAAGTATGACAACAGCCT | | 5'AGTCCTTCCACGATACCAAAGT | |
| mIFNLR1 | 5'CAGAAACGTGACACTCTTCTCC | | 5'CTGCACAATGCTCCACTGG | |
| mIL10RB | 5'ACCTGCTTTCCCCAAAACGAA | | 5'TGAGAGAAGTCGCACTGAGTC | |
| mMX1 | 5'GCATGGCTCAGGAGGTGG | | 5'GGATACACCAGGTTCCGC | |
| mGAPDH | 5'AGGTCGGTGTGAACGGATTTG | | 5'TGTAGACCATGTAGTTGAGGTCA | |
| IFNAR1 | 5'AACAGGAGCGATGAGTCTGTC | | 5'TGCGAAATGGTGTAAATGAGTC | |
| IFNAR2 | 5'ACCACTCCATTGTACCAACTCA | | 5'TGTGCTTCTCCACTCATCTGT | |
| IFNGR1 | 5'AGCGATTCCAGTATCCTCACT | | 5'CCAGGCTAAGCACTAGAAAGAGT | |
| IFNGR2 | 5'GCAGGCTTCCCAATGGATTTC | | 5'CCCGACAGTCACATTCCGATA | |
| HSV-1 ICP27 | 5'TTTCTCCAGTGCTACCTGAAGG | | 5'TCAACTCGCAGACACGACTCG | |
| HSV-1 UL30 | 5'CGCGCTTGGCGGGTATTAACAT | | 5'TGGGTGTCCGGCAGAATAAAGC | |
| HSV-1 VP16 | 5'TCGGCGTGGAAGAAACGAGAGA | | 5'CGAACGCACCCAAATCGACA | |
| HSV-1 UL36 | 5'CGCTGCACGAATAGCATGGAATC | | 5'CCAGCTCCCCGGAACACATTTA | |
| GFP | 5'AAGCTGACCCTGAAGTTCATCTGC | | 5'CTTGTAGTTGCCGTCGTCCTTGAA | |
| **McrBC nested PCR primers** | | Forward | | Reverse |
| First pair | | 5'CCAAAGTGCTGGGATTACAG | | 5'CTTACTCATCAGTAGAGGTGGG |
| Second pair | | 5'TCTCAGTGTGTTGCAAACACC | | 5'TGTCCACACTTTAGGGTCAAGT |
| **Bisulfite conversion sequencing primers** | | Forward | | Reverse |
| *CpG island I* | |  | |  |
| First pair | | 5'GTTTTATTGGGAAGTTATTGTTG | | 5'CCTCTACTTCATCTACACTTAA |
| Second pair | | 5'TTTAGGTGAATATTATAGTTTT | | 5'ACTAATAATCAATAACCCA |
| *CpG island II* | |  | |  |
| First pair | | 5'TTTTGGAGTATTGGATTGGT | | 5'TTCTCCAATTCTCCTAAAACT |
| Second pair | | 5'TTAAGTGTAGATGAAGTAGAGGT | | 5'TACCTATCCCAAAAACTC |
| **ChIP primers** | | Forward | | Reverse |
| IFNLR1 promoter | | 5'CCGGCCTTGAACTCTCCCT | | 5'GCGCTCGAAACTCGCCC |
| GAPDH promoter | | 5'TACGTCGGGGCCCACAC | | 5'GGAGGCTGCGGGCTCAAT |
| α-satellite region | | 5'CTGCACTACCTGAAGAGGAC | | 5'GATGGTTCAACACTCTTACA |
| Bax promoter | | 5'GTAGCTCATGCCTGTAATCC | | 5'GTCCAATCGCAGCTCTAATG |
| **EMSA oligonucleotides** | | Forward | | Reverse |
| Wild-type probe (-434~-401) | | 5'TAGATCCCGCCAATGGCATTGAGGCCGCGTAGCCAAA | | 5'TAGTTTGGCTACGCGGCCTCAATGCCATTGGCGGGAT |
| Mutant probe (-434~-401) | | 5'TAGATCCCGAACCGGGCATTGAGGCCGCGTAGCCAAA | | 5'TAGTTTGGCTACGCGGCCTCAATGCCCGGTTCGGGAT |
| Wild-type probe (-500~-467) | | 5'TAGGGGGCATTAAAGGGAATCGCGTGTGTAAGGCGC | | 5'TAGGCGCCTTACACACGCGATTCCCTTTAATGCCCC |
| Wild-type probe (-467~-434) | | 5'TAGGGAGCTCAGCATCCGGCTCAGAAACGCGCTCGG | | 5'TAGCCGAGCGCGTTTCTGAGCCGGATGCTGAGCTCC |
| MUT1 probe | | 5'TAGATCCAAAAAATGGCATTGAGGCCGCGTAGCCAAA | | 5'TAGTTTGGCTACGCGGCCTCAATGCCATTTTTTGGAT |
| MUT2 probe | | 5'TAGATCCCGCCAAAAAAATTGAGGCCGCGTAGCCAAA | | 5'TAGTTTGGCTACGCGGCCTCAATTTTTTTGGCGGGAT |
| MUT3 probe | | 5'TAGATCCCGCCAATGGCAAAAAGGCCGCGTAGCCAAA | | 5'TAGTTTGGCTACGCGGCCTTTTTGCCATTGGCGGGAT |
| MUT4 probe | | 5'TAGATCCCGCCAATGGCATTGAAAAAACGTAGCCAAA | | 5'TAGTTTGGCTACGTTTTTTCAATGCCATTGGCGGGAT |
| MUT5 probe | | 5'TAGATCCCGCCAATGGCATTGAGGCCGAAAAACCAAA | | 5'TAGTTTGGTTTTTCGGCCTCAATGCCATTGGCGGGAT |
| MUT6 probe | | 5'TAGATCCCGCCAATGGCATTGAGGCCGCGTAGAAAAA | | 5'TAGTTTTTCTACGCGGCCTCAATGCCATTGGCGGGAT |
